# A novel palmitic acid hydroxy stearic acid (5‐PAHSA) plays a neuroprotective role by inhibiting phosphorylation of the m‐TOR‐ULK1 pathway and regulating autophagy

**DOI:** 10.1111/cns.13573

**Published:** 2021-01-18

**Authors:** Jian‐tao Wang, Zhong‐yu Yu, Ying‐hong Tao, Ying‐chao Liu, Yan‐mei Wang, Qi‐lin Guo, Jian‐zhong Xue, Xiao‐hong Wen, Qian Zhang, Xiao‐die Xu, Cheng‐feng He, Wen‐jiao Xue, Jing‐chun Guo, Hou‐guang Zhou

**Affiliations:** ^1^ Department of Geriatric Neurology of Huashan Hospital National Clinical Research Center for Aging and Medicine Fudan University Shanghai China; ^2^ State Key Laboratory of Medical Neurobiology and MOE Frontiers Center for Brain Science Institutes of Brain Science Department of Translational Neuroscience Jing'an District Centre Hospital of Shanghai Fudan University Shanghai China; ^3^ Department of Medical Examination Center Huashan Hospital, Fudan University Shanghai China; ^4^ Department of Neurosurgery Provincial Hospital Affiliated to Shandong University Jinan China; ^5^ Department of Neurology Fifth Clinical Medical College of Yangzhou University Changshu Second People's Hospital of Jiangsu Province Changshu China

**Keywords:** 5‐PAHSA, autophagy, m‐TOR, oxidative stress, type‐2 diabetes mellitus

## Abstract

**Aims:**

Type 2 diabetes mellitus (T2DM) can lead to brain dysfunction and a series of neurological complications. Previous research demonstrated that a novel palmitic acid (5‐PAHSA) exerts effect on glucose tolerance and chronic inflammation. Autophagy was important in diabetic‐related neurodegeneration. The aim of the present study was to investigate whether 5‐PAHSA has specific therapeutic effects on neurological dysfunction in diabetics, particularly with regard to autophagy.

**Methods:**

5‐PAHSA was successfully synthesized according to a previously described protocol. We then carried out a series of *in vitro* and *in vivo* experiments using PC12 cells under diabetic conditions, and DB/DB mice, respectively. PC12 cells were treated with 5‐PAHSA for 24 h, while mice were administered with 5‐PAHSA for 30 days. At the end of each experiment, we analyzed glucolipid metabolism, autophagy, apoptosis, oxidative stress, cognition, and a range of inflammatory factors.

**Results:**

Although there was no significant improvement in glucose metabolism in mice administered with 5‐PAHSA, ox‐LDL decreased significantly following the administration of 5‐PAHSA in serum of DB/DB mice (*p* < 0.0001). We also found that the phosphorylation of m‐TOR and ULK‐1 was suppressed in both PC12 cells and DB/DB mice following the administration of 5‐PAHSA (*p* < 0.05 and *p* < 0.01), although increased levels of autophagy were only observed *in vitro* (*p* < 0.05). Following the administration of 5‐PAHSA, the concentration of ROS decreased in PC12 cells and the levels of CRP increased in high‐dose group of 5‐PAHSA (*p* < 0.01). There were no significant changes in terms of apoptosis, other inflammatory factors, or cognition in DB/DB mice following the administration of 5‐PAHSA.

**Conclusion:**

We found that 5‐PAHSA can enhance autophagy in PC12 cells under diabetic conditions. Our data demonstrated that 5‐PAHSA inhibits phosphorylation of the m‐TOR‐ULK1 pathway and suppressed oxidative stress in PC12 cells, and exerted influence on lipid metabolism in DB/DB mice.

## Graphical abstract

5‐PAHSA could activate autophagy in PC12 cells under diabetic condition via inhibition of m‐TOR‐ULK‐1 pathway, while this effect was not significant in diabetes animal models, and it has no significant impact on glucose metabolism in diabetes animals. More researches are needed to explore 5‐PAHSA’s metabolic effect.

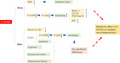

## 1 INTRODUCTION

The development of the global economy has led to significant changes in dietary structure. Consequently, there has been a substantial increase in the incidence of metabolic diseases over the last few years, particularly with regard to diabetes. According to the International Diabetes Federation (IDF), diabetes mellitus (DM), and complications related to DM cause more than 5. 0 million deaths per year. Type 2 diabetes mellitus (T2DM) has become one of the major risk factors threatening human health, both in developed and developing countries.^1^ The prevalence of T2DM has increased significantly over the past two decades, with an increased incidence of 4.8% annually between 2002 and 2012.^2^ These data indicate that T2DM will create a significantly greater health burden to the aging society.

Type 2 diabetes mellitus is associated with a range of complications; evidence shows that neurological complications are one of the most serious of these complications, including cognitive decline, white matter lesions, and depression.^3^, ^4^, ^5^ Of a wide range of neurological diseases, Alzheimer's disease (AD) appears to be the most common of the chronic complications. Research has shown that T2DM increases the risk of both AD and vascular dementia.^6^ Type 2 diabetes mellitus can also lead to blood‐brain‐barrier damage, axonal loss, and mitochondrial dysfunction.^7^ Furthermore, metabolic stress or inflammation‐induced insulin resistance of neurons in mice with T2DM will eventually lead to pathological amyloid deposition.^8^

The mechanisms underlying how hyperglycemia can exert effect on cognition have yet to be elucidated, although high levels of inflammatory factors and endoplasmic reticulum stress have been reported in T2DM mice models when fed a high‐fat diet (HFD).^8^ These findings suggest that inflammation maybe involved in the cognitive decline associated with diabetes. Oxidative stress has also been reported to become elevated in T2DM; intracellular reactive oxygen species (ROS) is known to induce neuronal injury and neurodegenerative disease.^9^ At present, there is no effective therapy for cognitive issues related to diabetes. Recent research has shown that intensive glycemia^10^ or blood pressure^3^ control has little effect on memory performance and may even give rise to complications such as hypoglycemia.^11^ Therefore, it is vital that we identify a new interventional method for diabetes and associated complications in the central nervous system.

Autophagy is a highly conserved homeostatic process that selectively degrades cellular components, including dysfunctional organelles and toxic proteins. The accumulation of dysfunctional proteins and organelles often leads to neuronal toxicity and neurodegeneration disease. Defects in autophagy are common in cases of T2DM.^12^ For example, microtubule associated protein 1 light chain 3 (LC3) II/I ratio was reduced in Goto‐Kakizaki (GK) rats (a model of hyperglycemia), while SQSTM1 was elevated.^13^ The impairment of autophagy can lead to neurotoxicity in diabetes.^13^ Abnormal levels of oxidative stress could also suppress autophagy via the TRPM2‐Ca^2+^‐CAMK2‐BECN1 pathway.^14^ Appropriate levels of autophagy are essential in the central nervous system as they are required for neuronal survival and cognition.^15^ Previous research has also shown that agonists of autophagy can play protective roles in diabetes. For example, a novel, natural product, named RG2, has been shown to activate autophagy *via* the unc‐51‐like kinase‐1(ULK‐1) and adenosine monophosphate‐activated protein kinase (AMPK) pathway and has also been shown to exert metabolic benefits in animal models of T2DM.^16^

As a suppressor of autophagy, mammalian target of rapamycin (m‐TOR) can be phosphorylated at the Ser2448 site via the PI3 K (Phosphatidylinositol 3‐kinase)/Akt signaling pathway. This inhibits autophagy by phosphorylating Ulk1 Ser 757 and by disrupting the interaction between ULK and AMPK.^17^ Inhibiting the phosphorylation of m‐TOR could promote autophagy and improve endothelial dysfunction in T2DM.^18^

According to Yore, et al,^19^ a novel compound, referred to as palmitic acid hydroxy stearic acid (PAHSA), was identified in mice that overexpressed Glut4 (AG40X). It has been reported that PAHSA shown promising effects on glucose tolerance and chronic inflammation.^19^ There are several isomers of PAHSAs; of these, 5‐PAHSA has been shown to be the most downregulated isomer in adipose tissue and the serum of insulin‐resistant mice. The metabolic effect of PAHSA has been confirmed, at least by a preliminary report. PAHSA exerts a metabolic effect on glucose homeostasis that is induced via the activation of G protein‐coupled receptors (GPRs) 40,^20^ and could also attenuate the immune response and promote β cell survival in a diabetic mice model.^21^ Furthermore, PAHSA also promoted the browning of white adipose tissue and suppressed the inflammatory pathway in animals without diabetes (OB/OB mice). However, very little is known about the effect of PAHSA on autophagy. We hypothesized that 5‐PAHSA supplementation may improve insulin resistance and complications related to diabetes. Thus, the aim of this study was to investigate whether 5‐PAHSA exerts specific therapeutic effects on neurological dysfunction in diabetes and to investigate the mechanisms underlying such effects, especially *via* the m‐TOR pathway.

## 2 METHOD AND MATERIALS

### 2.1 Synthesis of 5‐PAHSA

5‐PAHSA was synthesized in Shanghai Institute of Organic Chemistry, Chinese Academy of Sciences, in accordance with the method described previously by Yore, et al.^19^ Considering that the synthesis of new compounds is very expensive, we first synthesized only a small amount of 5‐PAHSA for our *in vitro* cell experiments. Once synthesized, 5‐PAHSA was dissolved in dimethyl sulfoxide (DMSO) (Sigma‐Aldrich, Darmstadt, Germany). Detailed procedure for synthesize of 5‐PAHSA was available in Supplementary Materials (SI).

### 2.2 Diabetic animal model grouping and 5‐PAHSA intervention

Following the acquisition of positive results from our *in vitro* experiments, we then continued to synthesize a new batch of 5‐PAHSA using the same method for all subsequent experiments. For *in vivo* experiments, 5‐PAHSA was dissolved in sodium carboxymethyl cellulose (China National Pharmaceutical Group Corporation). Male DB/DB mice (40 weeks‐of‐age) were used to create an animal model of type 2 diabetes^22^; male C57BL/6 mice of the same age were used as controls. Mice were maintained on a 12‐h light/dark cycle and had free access to food and water. Mice from each group were divided into 3 groups (*n* = 6–8 per group), including a vehicle group (sodium carboxymethyl cellulose), a low‐dose group (50 mg/kg 5‐PAHSA), and a high‐dose group (150 mg/kg 5‐PAHSA). Animals were administered with these doses once daily by oral gavage. Fasting blood glucose levels were measured before and at 10 and 30 days after administration. After 30 days of drug administration, the animals were sacrificed in order to harvest organs and collect blood samples. Three mice from each group were used for Western blotting; the others were used for immunohistochemistry and other experiments.

### 2.3 Measurement of fasting blood glucose in mice

Fasting blood glucose (FBG) was tested three times in each mouse: prior to the administration of 5‐PAHSA, and at 10 and 30 days after the administration of 5‐PAHSA. In order to avoid glycemic fluctuations, mice were placed in a safe and quiet environment; this practice avoided provoking the animals. Venous blood was collected by cutting the tail vein of each mouse at 9:00 am every time. Glucose levels were determined using Accu‐Check active bands and a glucometer (Roche Diagnostics, Basel, Switzerland). The oral glucose tolerance test (OGTT) was performed in C57BL/6 mice three days after 30 days of intervention. OGTT was performed at 9:00 am and was only performed after intervention.

### 2.4 Insulin and markers of inflammation and lipid metabolism

Blood samples were collected from each mouse after 30 days of treatment of 5‐PAHSA treatment. Animals were then sacrificed; the heart and the abdominal aorta were removed; blood samples were allowed to stand for 2 h at 4°C to separate serum and were then centrifuged at 1500 rpm for 20 min. Serum levels of C‐reactive protein (CRP), tumor necrosis factor‐α (TNF‐α), interleukin‐1α (IL‐1α), oxidized modified low‐density lipoprotein (ox‐LDL), and plasma insulin were all measured by ELISA kits in accordance with the manufacturer's protocols (Abcam, Cambridge, UK).

### 2.5 Creating a cell model of diabetes *in vitro*

Highly differentiated PC12 cells (Shanghai Fuheng Biology, 5th generation, Shanghai, China) were used as neuron for all *in vitro* cell model experiments.^23^ We created a diabetic environment *in vitro* by adding high concentrations of glucose and fatty acids. Cells were cultured separately in 4 types of medium, including Dulbecco's modified eagle medium (DMEM) high glucose (Tianjin Hao Yang Biological Manufacture cooperation, Tianjin, China), DMEM with extra glucose and fatty acid (GF, glucose 100 mmol/L, fatty acid 250 μmol/L), GF + DMSO, and GF+5‐PAHSA (30 μmol/L). 5‐PAHSA was dissolved in DMSO (Sigma‐Aldrich) at a concentration of 90 mmol/L; the working stock solution of 5‐PAHSA was diluted in medium at a final concentration of 30 μmol/L. Cells were cultured in an incubator (Thermo Fishier, Waltham, USA) at 37°C and 5% CO_2_ for 24 h. After treatment, PC12 cells were collected and lysed in order to extract proteins.

### 2.6 Detection of reactive oxygen species (ROS)

Fluorescent probes (Beyotime Technology, Shanghai, China) were diluted in serum‐free medium to a final concentration of 10 μmol/L. PC12 cells were then cultured in 96‐well plates and divided into two groups (GF+DMSO and GF+5‐PAHSA), as previously above. After 24 h of intervention, the culture media were removed and the fluorescent probes were added. After 20 min of incubation at 37°C and 5% CO_2_, the cells were washed three times in serum‐free medium. Finally, fluorescence intensity was detected with a fluorescence microplate reader (Bio Tek Synergy H4, Winooski, USA).

### 2.7 Y‐maze experiment

After 30 days of 5‐PAHSA administration, we used the Y‐maze test^24^ to investigate the cognitive function of DB/DB mice and C57BL/6 mice. Y‐maze test could assess short‐term memory of mice, which was the initial symptom of AD. The Y‐maze consisted of three identical arms (Figure 6(A)), and the experiment includes two phases. The first stage was a training phase and involves mice being trained to alternately enter three arms (eg, 1, 2, 3 or 3, 1, 2). The second phase was an experiment phase. Mice were randomly placed into one of the arms; the total sequence of mice entering into the arms, along with the correct sequences, was recorded. The final parameter (which reflects the cognitive ability of the mice) was calculated as follows: (correct sequences/total sequences) × 100%.

### 2.8 Western blotting

Proteins from cells and experimental mice (3 cortexes from each group) were extracted with radioimmunoprecipitation assay (RIPA) lysis buffer (Epizyme Biotech, Shanghai, China). Proteins were then incubated with loading buffer (Epizyme Biotech) at a temperature of 100°C for 10 min. Equal amounts of protein (10 μg/lane) from each sample were then separated by 7.5% or 12.5% SDS‐polyacrylamide gel electrophoresis and transferred to a polyvinylidene fluoride (PVDF) membrane (Millipore, Darmstadt, Germany). Membranes were blocked with 5% non‐fat dried milk (non‐phosphorylated antibody) or bovine serum albumin (phosphorylated antibody) and then incubated overnight at 4°C with antibodies to determine the levels of the following proteins: phosphorylation m‐TOR Ser2448 (Cell‐Signal Technology, Boston, USA); phosphorylation ULK1 Ser 757; PI3 Kinase Class III (Cell‐Signal Technology); SQSTM1/p62 (Cell‐Signal Technology); beclin‐1(Cell‐Signal Technology); BCL‐2 associated X protein (BAX) (Cell‐Signal Technology); Cleaved Caspase‐3 (Cell‐Signal Technology); and LC3B (Sigma‐Aldrich). The structural protein, β‐actin (Cell Signaling Technology), was used to normalize protein loading. The following morning, membranes were incubated with specific secondary antibodies (Cell‐Signal Technology) for 1 h. Peroxidase activity was then visualized with a Femto Light Chemiluminescence Kit (Epizyme Biotech). Protein levels were then quantified using Image Lab software (Bio‐Rad, Hercules, USA).

### 2.9 Statistical analysis

All data were analyzed using Prism 8.3.0 software (GraphPad Software, San Diego, USA) and expressed as mean ± standard error of the mean (SEM). Continues data of each group were tested for normality prior to analysis. Analysis of variance (ANOVA) was used to test normally distributed data for differences among multiple groups, and post hoc pairwise comparison was conducted when significant difference was observed. The Kruskal‐Wallis test (*H* test) was used for data that were not normally distributed. To compare differences between two groups, we used the *t* test for data that were normally distributed, and the Mann‐Whitney *U* test was used for data that were not normally distributed. Differences were considered to be statistically significant at *p* < 0.05. Detailed ANOVA table was available in SI.

## 3 RESULTS

### 3.1 5‐PAHSA was successfully synthesized with high purity and passed molecular structure validation

**FIGURE 1:**
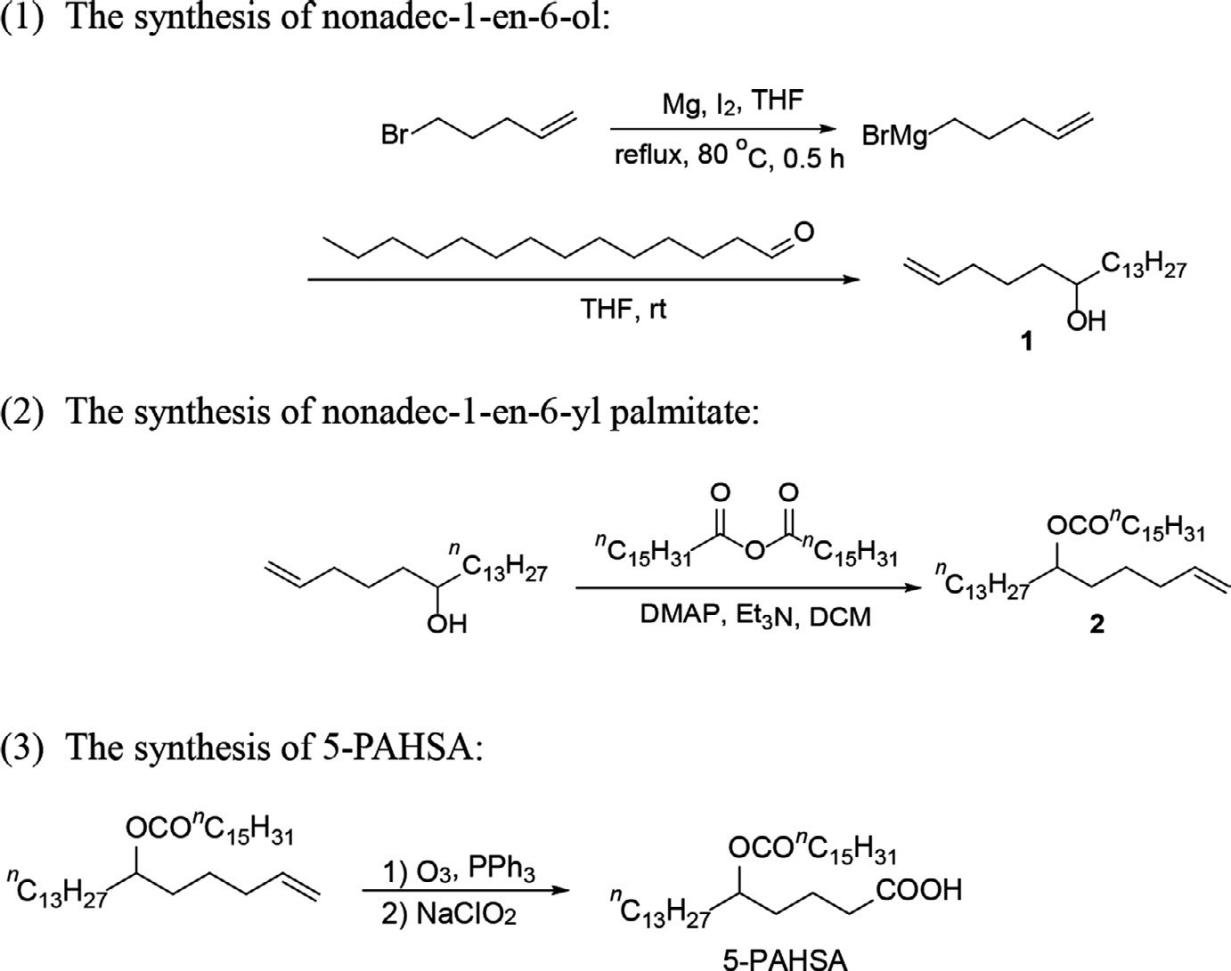
The brief procedure for synthesis of 5‐PAHSA

5‐PAHSA was synthesized according to a procedure reported by Kahn and co‐workers.^19^ The synthetic route is shown in Figure 1. The addition of the Grignard reagent, which was used immediately after its preparation, to tetradecanal gave nonadec‐1‐en‐6‐ol (compound **1**). After this alcohol was converted into the ester **2**, the double bond was first oxidized by O_3_to generate an aldehyde group and the further oxidation by NaClO_2_ afforded the final product, 5‐PAHSA. Pure 5‐PAHSA was isolated by flash column chromatography. Its purity and structure were determined by ^1^H NMR spectroscopy (Please see SI).

### 3.2 Fasting blood glucose, glucose tolerance, and plasma insulin did not improve significantly after 30 days of 5‐PAHSA administration; however, ox‐LDL was significantly lower in DB/DB mice

**FIGURE 2:**
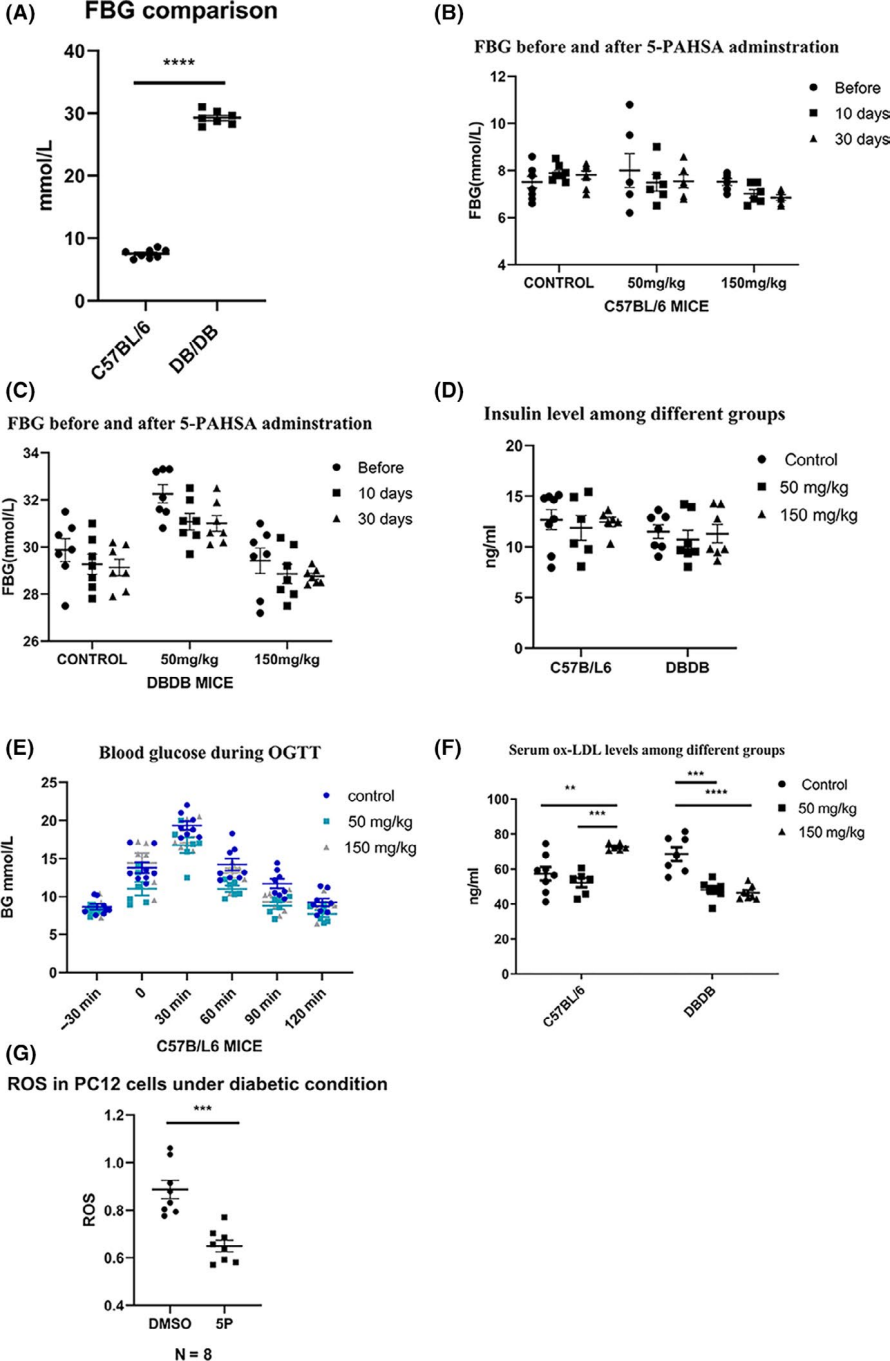
Glucolipid metabolism of mice after 10 and 30 days 5‐PAHSA administration and ROS in PC12 cells after 5‐PAHSA administration. (A) Fasting blood glucose of C57BL/6 and DB/DB mice before 5‐PAHSA administration. (B‐C) Fasting blood glucose of C57BL/6(B) and DB/DB(C) mice. (D) Plasma insulin of DB/DB and C57BL/6 mice. (E) OGTT test of C57BL/6 mice 3 days after 5‐PAHSA administration. (F) Plasma ox‐LDL of DB/DB and C57BL/6 mice. (G) ROS in PC12 cells. Data are mean ± SEM. *n* = 6–8 for A–F, *n* = 8 for G. *p < 0.05, **p < 0.01, ***p < 0.001, ****p < 0.0001 (unpaired *t* test: A and G, ANOVA and Kruskal‐Wallis test: B–F)

Low and high doses of 5‐PAHSA were administered to C57BL/6 and DB/DB mice (n = 6–8 per group). We then tested FBG before 5‐PAHSA administration and at 10 and 30 days after 5‐PAHSA treatment. We found that the levels of FBG were significantly elevated in DB/DB mice (*p* < 0.0001) (Figure 2(A)) and slightly lower in both the C57BL/6 and DB/DB mice after 5‐PAHSA treatment, although this difference was not statistically significant (Figure 2(B)–(C)). Levels of plasma insulin had no significant difference between C57BL/6 and DB/DB mice and remained unchanged in both the DB/DB and C57BL/6 mice after low‐ and high‐dose 5‐PAHSA treatment (Figure 2(D)). There was also no significant improvement in C57BL/6 mice with regard to the OGTT test, thus showing that glucose tolerance had not been improved (Figure 2(E)). However, we also found that the serum levels of ox‐LDL in the DB/DB mice were significantly lower after 30 days of 5‐PAHSA treatment (*p* < 0.001 in the low‐dose group; *p* < 0.0001 in the high‐dose group), and increased in C57BL/6 high‐dose group (*p* < 0.01) (Figure 2(F)), thus suggesting that 5‐PAHSA might exert effects on lipid metabolism and the suppression of lipid peroxidation in DB/DB mice but not in C57BL/6 mice.

### 3.3 Levels of reactive oxygen species in PC12 cells were decreased following 5‐PAHSA administration

Compared with the vehicle (DMSO) groups, the levels of ROS in PC12 cells treated with 5‐PAHSA were significantly lower (n = 8 for each group, *p* < 0.001) (Figure 2(G)), thus demonstrating that 5‐PAHSA could reduce oxidative stress in neurons under diabetic environments.

### 3.4 Levels of autophagy in neurons under diabetic conditions were elevated by inhibiting the phosphorylation of m‐TOR in PC12 following 5‐PAHSA administration

**FIGURE 3:**
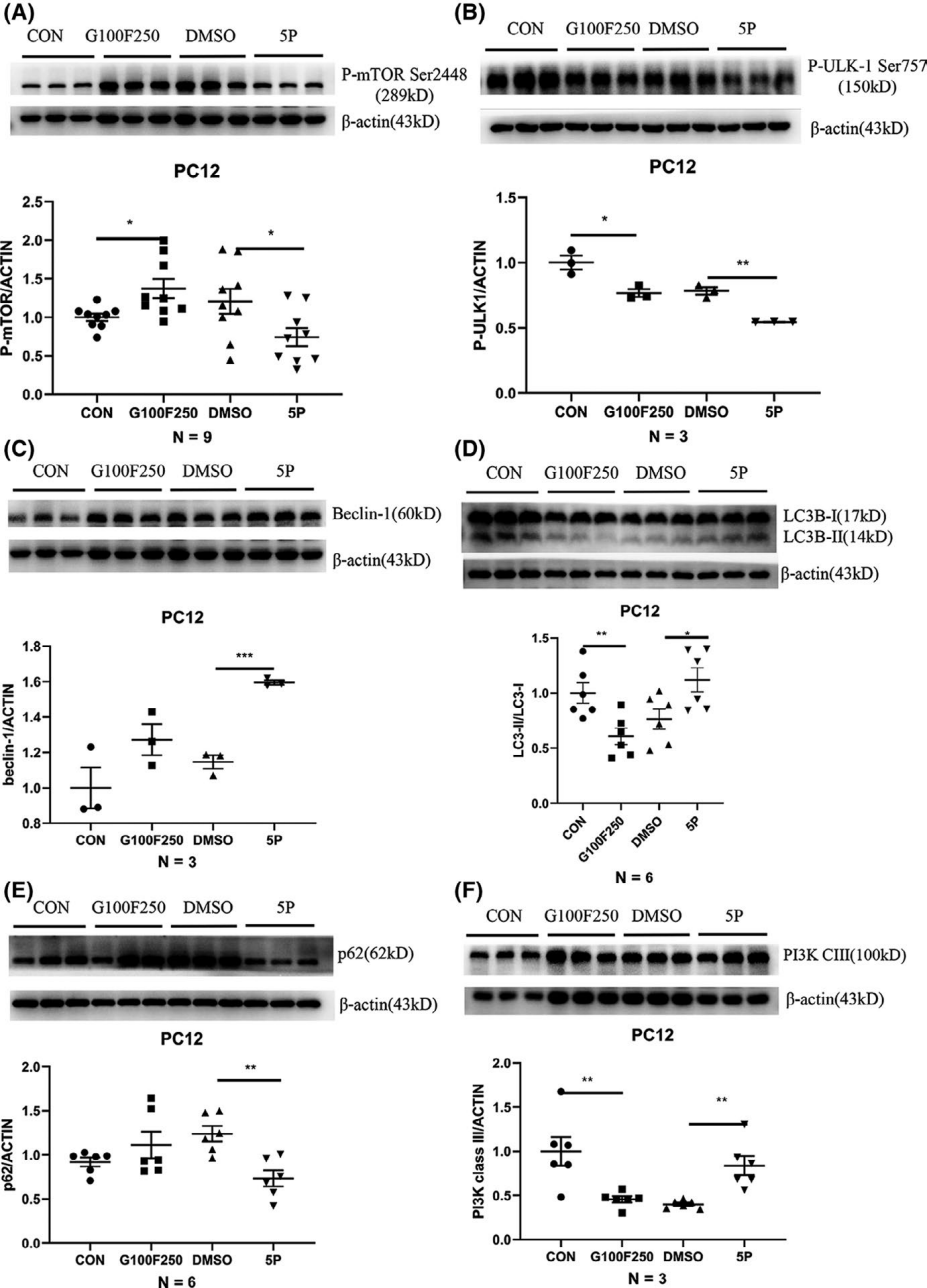
Autophagy‐related proteins in PC12 cells after 24 h 5‐PAHSA treatment. Phosphorylation of m‐TOR at Ser2448 (A), Phosphorylation of ULK‐1 at Ser757 (B), beclin‐1 (C), LC3B II/I (D), SQSTM/p62 (E), PI3 K class III (F). Data are mean ± SEM. *n* = 9 for A, *n* = 6 for D and E, *n* = 3 for B, C and F. *p < 0.05, **p < 0.01, ***p < 0.001 (A–F: unpaired *t* test)

Autophagy may play a protective role in diabetic individuals. We hypothesized that 5‐PAHSA could counteract the effect of high glucose and free fatty acid conditions in neurons by enhancing the levels of autophagy. Compared with the vehicle group (DMSO), the 5‐PAHSA group showed a significant reduction in the phosphorylation of m‐TOR at Ser2448 (*p* < 0.05, *n* = 9 for each group) (Figure 3(A)). The phosphorylation of ULK1 at Ser757 was also decreased (*p* < 0.01, *n* = 3 for each group) (Figure 3(B)) under diabetic conditions. Other proteins that are positively associated with autophagy, such as beclin‐1, LC3B II/I ratio, and PI3K class III, were also elevated in response to 5‐PAHSA treatment; the levels of SQSTM/p62 were significantly reduced (*p* < 0.001 for beclin‐1, *p* < 0.01 for SQSTM/p62 and PI3K class III, and *p* < 0.05 for LC3II/I ratio; *n* = 6 for LC3 and SQSTM/p62; *n* = 3 for beclin‐1 and PI3K) (Figure 3(C)–(F)). These changes demonstrated enhanced levels of autophagy in PC12 cells after 5‐PAHSA treatment in diabetic conditions.

### 3.5 The phosphorylation of m‐TOR at Ser2448 was suppressed in the cortex of DB/DB mice after 30 days of 5‐PAHSA administration

**FIGURE 4:**
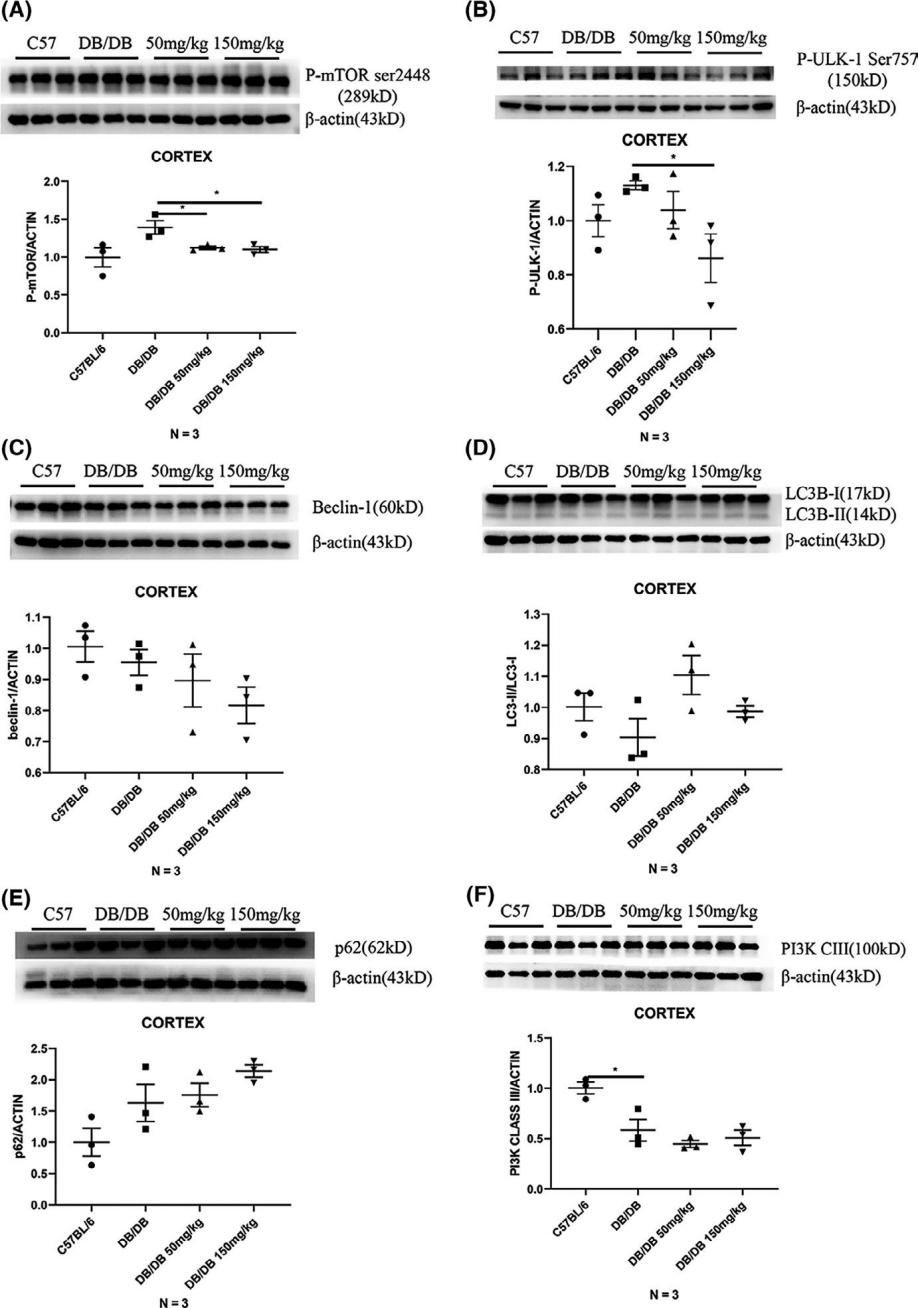
Autophagy‐related proteins of mice's cortex after 30 days 5‐PAHSA administration. Phosphorylation of m‐TOR at Ser2448 (A), Phosphorylation of ULK‐1 at Ser757 (B), beclin‐1 (C), LC3B II/I (D), SQSTM/p62 (E), PI3 K class III (F). Data are mean ± SEM. *n* = 3, *p < 0.05, **p < 0.01, ***p < 0.001 (A–F: unpaired *t* test)

We determined the levels of autophagy‐related proteins in the cortex of both C57BL/6 and DB/DB mice after 30 days of 5‐PAHSA administration (*n* = 3 per group). We found that compared with C57BL/6 mice, the phosphorylation of m‐TOR at Ser2448 was slightly elevated in the DB/DB mice and that both low and high doses of 5‐PAHSA could suppress this phosphorylation (*p* < 0.05) (Figure 4(A)), as could UKL‐1 Ser 757 (Figure 4(B)). Other autophagic indicators were not changed significantly after low‐ or high‐dose 5‐PAHSA administration, including beclin‐1, LC3BII/I ratio, p62, and PI3K class III (Figure 4(C)–(F)). These results demonstrated that 5‐PAHSA could suppress phosphorylation of the m‐TOR‐ULK1 pathway in the cortex of DB/DB mice but could not significantly induce autophagic flux.

### 3.6 The levels of apoptosis‐related proteins did not decrease significantly in PC12 cells when treated with 5‐PAHSA

**FIGURE 5:**
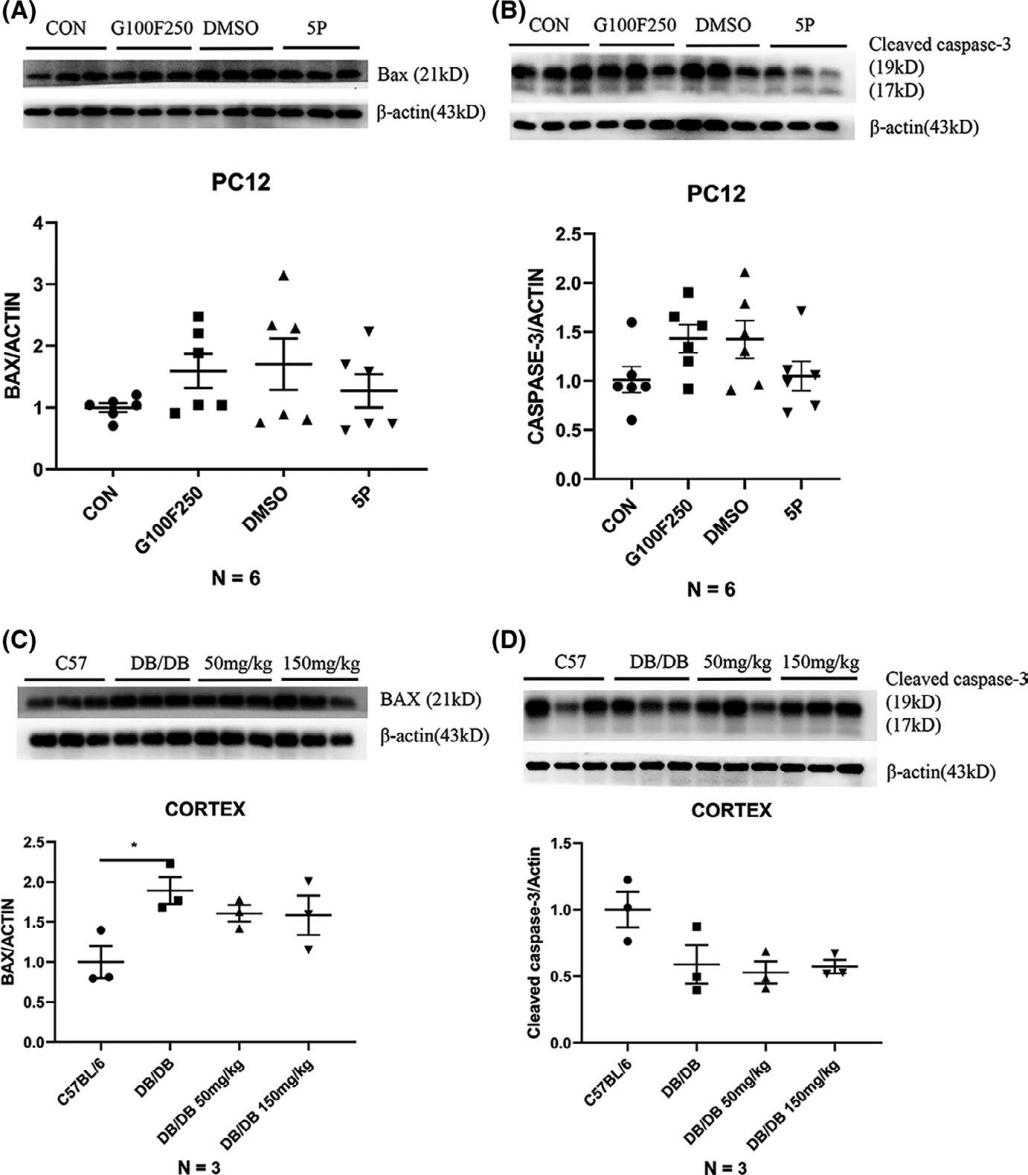
Apoptosis‐related protein in PC12 cells after 24 h 5‐PAHSA administration and in mice after 30 days 5‐PAHSA administration. (A–B) BAX and Cleaved caspase‐3 in PC12 cells. (C–D) BAX and Cleaved caspase‐3 in mice. Data are mean ± SEM. *n* = 6 for A and B, n − 3 for C and D. *p < 0.05, **p < 0.01, ***p < 0.001 (A–F: unpaired *t* test)

Next, we detected the levels of apoptosis‐related proteins, such as BAX and Cleaved caspase‐3, in PC12 cells in order to explore the neuroprotective effects of 5‐PAHSA. The levels of BAX and Cleaved caspase‐3 were slightly increased under diabetic conditions and showed a decreasing trend after 5‐PAHSA administration; however, these changes were not statistically significant (*n* = 6) (Figure 5(A)–(B)).

### 3.7 Apoptosis‐related proteins did not change after 30 days of 5‐PAHSA administration in both C57BL/6 and DB/DB mice

Next, we detected the levels of apoptosis‐related proteins, such as BAX and Cleaved caspase‐3, in the brains of both C57BL/6 and DB/DB mice. There were no significant differences when comparing the vehicle, low‐, and high‐dose groups (*n* = 3) (Figure 5(C)–(D)). This result showed that 5‐PAHSA did not directly affect apoptosis in the mouse brain. Data also showed that 5‐PAHSA did not significantly affect the levels of other autophagy proteins, except for m‐TOR.

### 3.8 The cognition of both C57BL/6 and DB/DB mice was not significantly elevated after 30 days 5‐PAHSA administration

**FIGURE 6:**
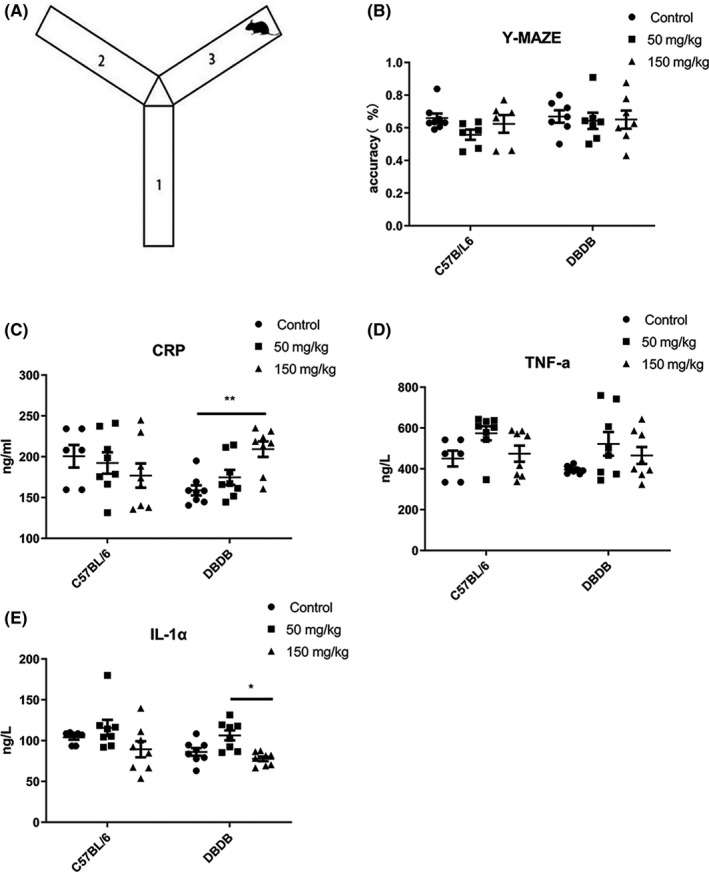
Y‐maze experiment and inflammatory factors in serum of mice after 30 days 5‐PAHSA administration. (A) Schematic illustration of Y‐maze test. (B) Y‐maze test result. (C–E) Inflammatory factors: In all of 3 inflammatory factors: CRP (C), TNF‐α (D) and IL‐1α (E). Data are mean ± SEM. *n* = 6–8, *p < 0.05, **p < 0.01, (ANOVA and Kruskal‐Wallis test)

Y‐maze test for each group was conducted after 30 days administration (*n* = 6–8 for each group). The number of times that mice entered the Y‐maze arms in the correct order was counted. There was no significant difference between vehicle groups and low‐ and high‐dose groups of both kinds of mice (Figure 6(B)). This result suggested that no significant improvements on spatial memory were observed in both C57BL/6 and DB/DB mice after 5‐PAHSA administration.

### 3.9 Inflammatory factors CRP was increased in high‐dose DB/DB mice, and others were remained unchanged after 5‐PAHSA administration

Serum inflammatory factors were tested for all six 6 groups of mice in order to assess the safety of using relatively high doses of 5‐PAHSA. Our data showed that CRP increased in high‐dose DB/DB mice but remained unchanged in other groups (Figure 6(C)). Other inflammatory factors, such as TNF‐α and IL‐1α, did not change significantly with increased dosage (Figure 6(D)–(E)). These results suggest that a chronic treatment involving 5‐PAHSA did not result in obvious dose‐dependent chronic inflammation.

## 4 DISCUSSION

The first publication to describe PAHSA referred to the fact that this drug could reduce blood glucose levels and insulin resistance.^19^ Since then, a multitude of studies have investigated the potential role of PAHSA in a wide range of disease states. Studies have shown that PAHSA plays a beneficial role in metabolic events by activating G protein‐coupled receptor 40(GPR40).^20^ PAHSA is also known to suppress lipolysis and enhance insulin sensitivity in the liver and white adipose tissue^25^ and can also promote β‐cell survival by attenuating the immune response.^21^ However, there is contradictory evidence in the existing literature. For example, Pflimlin, et al. reported the administration of 5‐PAHSA and 9‐PAHSA administration had no significant effect on blood glucose and insulin secretion when compared between vehicle and PAHSA groups in four types of HFD‐induced T2DM mice.^26^

In this study, there was no significant effect on glucose metabolism and insulin secretion after 10 and 30 days of intervention with 5‐PAHSA for both DB/DB and C57BL/6 mice. Furthermore, the administration of 5‐PAHSA had no effect on OGTT tests in C57BL/6 mice. This implied that a reduction in glucose level, or an improvement in insulin sensitivity, might not be the only mechanism exerted by PAHSA. Furthermore, the excessive blood glucose levels of DB/DB mice might only represent one possible reason for this phenomenon. These findings were consistent with those described by Pflimlin, et al^26^ and highlighted the fact that further research needed to be carried out to investigate the specific mechanisms involved within the action of 5‐PAHSA on metabolic events.

Insulin resistance is often accompanied by autophagy impairment. Lipocalin‐2 has been shown to inhibit autophagy and induce insulin resistance in H9c2 cells.^27^ Insulin resistance and hepatic steatosis were also improved in T2DM rats when treated with the autophagy modulator rapamycin.^28^ Considering the effect of 5‐PAHSA on the enhancement of autophagy, reduced levels of insulin resistance in peripheral organs via enhancement in autophagy may be a critical aspect of 5‐PAHSA’s metabolic benefits; this hypothesis is consistent with a previous study of PAHSA on hepatic and systemic insulin sensitivity.^25^

We also made an interesting discovery in the present study. Both low and high doses of 5‐PAHSA could significantly inhibit the abnormal increase of ox‐LDL in the serum of DB/DB mice, thus suggesting that 5‐PAHSA may have a promising effect on lipid metabolism in diabetes and provide a novel approach for treating diabetic complications. Previous research found that quercetin could reduce HFD‐induced ox‐LDL accumulation in the liver by enhancing autophagy.^29^ We therefore hypothesize that metabolic effects of 5‐PAHSA on ox‐LDL potentially occur via the enhancement of autophagy in peripheral tissues, such as the liver. Besides, ox‐LDL was proved to have pro‐atherosclerosis effect in cardiovascular disease, removal of ox‐LDL has shown promising effect,^30^ which demonstrating that anti‐atherosclerosis maybe involved in 5‐PAHSA’s metabolic benefit. Neuro‐inflammation was one of the mechanisms in AD and diabetic‐neurodegeneration,^31^ considering the pro‐inflammation of ox‐LDL, 5‐PAHSA may plays a neuroprotective role via anti‐inflammation. Certainly, further research is now needed to confirm this hypothesis. ox‐LDL was elevated in high‐dose C57BL/6 mice, suggesting that high‐dose 5‐PAHSA may disturb lipid metabolism in healthy mice models.

Autophagy is a critical process for metabolic homeostasis but is impaired in T2DM. Previous research showed that autophagy was suppressed in the hippocampus of the GK diabetic model in rats and that high glucose levels could impair autolysosome synthesis.^13^ In HFD‐induced zebrafish models of T2DM, autophagy‐related genes, including *Atg3*, *Atg4*B, *Atg7*, and *Foxo3*, were all shown to be expressed at low levels; levels of the autophagy inhibitor gene m‐TOR were increased, and downstream insulin signals were impaired.^32^ Defective autophagy can also increase ER stress.^33^ In the present study, reduced levels of autophagy were also observed in both PC12 cells and mice under diabetic conditions, although there were no statistically significant relationships observed in the diabetic mice; this may be due to the relatively small sample size for protein detection.

Several autophagy agonists have been used for the treatment of T2DM, especially with regard to reducing the activity of the m‐TOR pathway signaling. For example, Liraglutide has been shown to reduce blood glucose levels and improve hepatic lipase activity,^34^ and Ghrelin has also been shown to attenuate lipotoxicity, fibrosis, and inflammation response.^35^ Other research has shown that the phosphorylation of ULK‐1 was also reduced when autophagy was enhanced in diabetic nephropathy.^36^ In the present study, we found that the administration of 5‐PAHSA reduced the phosphorylation of m‐TOR in both PC12 cells under diabetic condition and DB/DB mice. However, autophagy was only activated in PC12 cells, not DB/DB mice. Two reasons may underlie these findings. First, the activation of autophagy is a chronic process and a 30‐day interventional period may not be sufficient to induce obvious autophagic flux. Secondly, our sample size was relatively small.

Previous studies have confirmed that appropriate levels of autophagy in the brain are essential for maintaining cognitive function. It has been reported that the accumulation of Aβ leads to an increase in m‐TOR signaling and that a downregulation of m‐TOR can slow the progression of AD and extend life span in transgenic mouse models of AD.^37^, ^38^ Autophagy also plays an important role in T2DM‐related neurodegeneration. Regulators of autophagy, such as G‐CSF, have been shown to exert neuroprotective effects in aged diabetic mice. In the previous study, we found that G‐CSF significantly enhanced the levels of beclin‐1 and LC3 II/I ratio in DB/DB mice and reversed increases in the levels of NF‐κB, spatial memory, cognition was improved and the hippocampal atrophy of DB/DB mice were significantly ameliorated.^39^ In diabetes, cerebral metabolic inflammation is associated with intracellular stress and autophagic deficit; the suppression of autophagy could induce proinflammatory effects via oxidative stress and the NF‐κB pathway.^40^

*In Vitro* experiments showed that levels of autophagy were elevated when we inhibited the phosphorylation of m‐TOR by 5‐PAHSA in PC12 cells. Given this information, we hypothesize that 5‐PAHSA activated autophagy by suppressing the phosphorylation of the m‐TOR signaling pathway. In our *in vivo* experiments, 5‐PAHSA suppressed the phosphorylation of m‐TOR but did not activate autophagy in the cortex of DB/DB mice; furthermore, there was no reduction in the levels of apoptosis‐related proteins, and no improvement in performance in the Y‐maze. These results demonstrate that there was no significant improvement in neuroprotection or cognition without the activation of autophagy. Only 3 cortexes were used for Western blotting from each experimental group; it is possible that an insufficient sample size led to these non‐significant differences. It is also possible that 40‐week‐old C57BL/6 mice and DB/DB mice may not be old enough to develop cognitive dysfunction, so we observed no obvious differences with regard to cognitive function. Similar with the activation of autophagy, the cognitive decline of DB/DB mice is also a chronic pathophysiological process; it is possible that the 30 days intervention period was not sufficient for the enhancement of autophagy and cognitive improvement in the brain.

Levels of free radicals, such as ROS, are known to be high in T2DM due to abnormal glucose metabolism.^41^ Abnormal levels of oxidative stress in diabetes can cause many complications. For example, the dysfunctional activity of pancreatic beta cells in diabetes is often accompanied by oxidative stress.^42^ Alzheimer's disease in diabetic patients is also known to be related to excessive levels of oxidative stress^43^; anti‐oxidative stress therapy can help ameliorate cognitive decline.^44^ In this study, the administration of 5‐PAHSA was found to activate autophagy and reduce levels of ROS in PC12 cells under diabetic conditions. These results demonstrated that 5‐PAHSA could play a neuroprotective role *via* two different pathways: autophagy and anti‐oxidative stress.

As two types of programmed cell death, the interaction between autophagy and apoptosis is complex. Autophagy usually precedes apoptosis.^45^ Enhanced levels of autophagy could suppress apoptosis by several methods, for example, by removing BAX, BCL‐2 antagonists, or killer (BAK).^46^ In this study, the slightly reduced levels of apoptosis after 5‐PAHSA administration were accompanied by enhanced levels of autophagy, although there were no statistically significant differences. This suggested that the potential ability of 5‐PAHSA to provide neuroprotective effects maybe independent of anti‐apoptosis.

The inflammatory response is involved in T2DM. T2DM is a chronic inflammatory disease that is combined with insulin resistance and beta cell failure. Inflammatory factors are known to be elevated in T2DM individuals, including CRP and interleukin‐6 (IL‐6).^47^, ^48^ Previous study reported that 9‐PAHSA has been shown to reduce inflammatory responses in adipose tissue by inhibiting the NF‐kappa B pathway.^49^ In our present study, no significant differences were observed among C57BL/6 and DB/DB mice. Thirty days of 5‐PAHSA treatment increased CRP in high‐dose DB/DB mice, which means that 150 mg/kg may induce mild inflammatory response in DB/DB mice. But 5‐PAHSA did not induce significant changes in other inflammatory factors such as IL‐1α and TNF‐α in either C57BL/6 or DB/DB mice. Besides, exorbitant isoforms of CRP could block autophagy in zebrafish models,^50^ which may explain the relatively low levels of autophagy in DB/DB mice after 5‐PAHSA administration. This implies that the proper dosage of 5‐PAHSA was worthy to be further explored.

This study has several strengths. First, we successfully synthesized 5‐PAHSA by ourselves with good levels of purity. Previous studies of PAHSA focused on its effect on glucose metabolism or inflammation. Our study revealed the neuroprotective effects of 5‐PAHSA from the perspective of enhancing autophagy. Our research demonstrated that 5‐PAHSA exerted a promising and potential effect on enhancing autophagy in an animal model of diabetes. Consequently, more studies are now needed to investigate the specific mechanisms and pathways underlying the effects of 5‐PAHSA on diabetes and associated complications in the central nervous system. Furthermore, 5‐PAHSA was shown to reduce serum levels of ox‐LDL levels in DB/DB mice, thus indicating that the regulation of lipid metabolism may become a novel perspective for exploring the metabolic benefits of 5‐PAHSA in the future. Furthermore, we found that 5‐PAHSA could also reduce the levels of ROS in PC12 cells under diabetic conditions; this has not been reported previously. Collectively, these results suggested that 5‐PAHSA might have potential neuroprotective effects under diabetic conditions.

There were several limitations to our research that need to be considered when interpreting our findings. Firstly, the levels of autophagy were not elevated in the brains of our experimental mice; this may have been due to our relatively small sample size and the short duration of 5‐PAHSA administration. Furthermore, we were unable to identify the potential effect of 5‐PAHSA on insulin resistance. We did not test the levels of autophagy in peripheral tissue in an attempt to explain the reduction of ox‐LDL in DB/DB mice. Further researches are needed to address these remaining issues. We need to explore the effect of 5‐PAHSA on the cortex of DB/DB mice with a much larger sample size and further explore the effect of 5‐PAHSA on apoptosis and oxidative stress in neurons under T2DM conditions. We also need to confirm the metabolic effects of 5‐PAHSA on insulin sensitivity and autophagy in the liver and adipose tissue, and proper dosage for DB/DB mice. We will also explore 5‐PAHSA’s permeability of blood‐brain‐barrier in future. These studies should help us to elucidate the downstream signaling pathways of m‐TOR that are inhibited by 5‐PAHSA; this will help us to confirm the role of 5‐PAHSA in the enhancement of autophagy.

## 5 CONCLUSION

This study demonstrated the effect of 5‐PAHSA on the enhancement of autophagy by inhibiting the phosphorylation of m‐TOR and by reducing the levels of ROS in PC12 cells under diabetic conditions. We also found that 5‐PAHSA could reduce the levels of serum ox‐LDL in models of diabetes. There were no significant improvements in either C57BL/6 or DB/DB mice with regard to glucose tolerance or insulin secretion. The phosphorylation of m‐TOR was suppressed in the cortexes of DB/DB mice although levels of autophagy were not elevated after the administration of 5‐PAHSA. These effects of 5‐PAHSA provided us with a novel approach to manage diabetic neurodegenerative diseases; it should be possible to regulate autophagy by inhibiting the phosphorylation of m‐TOR in neurons. We speculate that the metabolic effects of 5‐PAHSA do not directly cause a reduction of blood glucose or the stimulation of insulin secretion; further research is required to fully investigate the mechanisms involved.

## CONFLICT OF INTEREST

The authors state no potential conflicts of interest for the research, authorship, and publication of this article.

## ETHICAL STATEMENT

This study was proved by Animal Welfare and Ethics Group, Department of Laboratory Animal Science, Fudan University (2020‐Huashan hospital‐JS190).

## Supporting information

Supplementary Material

Supplementary Material

Supplementary Material

## Data Availability

The data that supports the findings of this study are available in the supplementary material of this article.
