# Supplementary material for: A novel palmitic acid hydroxy stearic acid (5‐PAHSA) plays a neuroprotective role by inhibiting phosphorylation of the m‐TOR‐ULK1 pathway and regulating autophagy
Source: CNS Neurosci Ther. 2021 Jan 18;27(4):484–96. doi: 10.1111/cns.13573 (PMC7941174; doi:10.1111/cns.13573)
Supplement: Supplementary file 2 — Supplementary Material [file CNS-27-484-s003.pdf]

FBG C57

BEFORE

ANOVA table	SS	DF	MS	F (DFn, DF	P value
Treatment (between columns)	0.6758	2	0.3379	F (2, 21) =	P=0.3166
Residual (within columns)	5.838	21	0.278		
Total	6.513	23			

10 DAYS

ANOVA table	SS	DF	MS	F (DFn, DF	P value
Treatment (between columns)	0.9744	2	0.4872	F (2, 15) =	P=0.7181
Residual (within columns)	21.58	15	1.439		
Total	22.56	17			

30 DAYS

ANOVA table	SS	DF	MS	F (DFn, DF	P value
Treatment (between columns)	0.6744	2	0.3372	F (2, 15) =	P=0.0818
Residual (within columns)	1.702	15	0.1134		
Total	2.376	17			

FBG DB/DB

BEFORE

ANOVA table	SS	DF	MS	F (DFn, DF	P value
Treatment (between columns)	2.175	2	1.088	F (2, 18) =	P=0.4407
Residual (within columns)	22.82	18	1.268		
Total	25	20			

10 DAYS

ANOVA table	SS	DF	MS	F (DFn, DF	P value
Treatment (between columns)	6.98	2	3.49	F (2, 18) =	P=0.0411
Residual (within columns)	16.39	18	0.9106		
Total	23.37	20			

No differernce in mutiple comparison with control

30 DAYS

ANOVA table	SS	DF	MS	F (DFn, DF	P value
Treatment (between columns)	1.755	2	0.8776	F (2, 18) =	P=0.4694
Residual (within columns)	20.02	18	1.112		
Total	21.78	20			

INSULIN

C57

ANOVA table	SS	DF	MS	F (DFn, DF	P value
Treatment (between columns)	7.328	2	3.664	F (2, 18) =	P=0.6194
Residual (within columns)	134.1	18	7.449		
Total	141.4	20			

DB/DB

ANOVA table	SS	DF	MS	F (DFn, DF	P value
Treatment (between columns)	9.658	2	4.829	F (2, 17) =	P=0.2787
Residual (within columns)	59.54	17	3.502		
Total	69.2	19			

OGTT (min)

-30

ANOVA table	SS	DF	MS	F (DFn, DF	P value

Treatment (between columns)	0.7916	2	0.3958	F (2, 17) =	P=0.6741
Residual (within columns)	16.67	17	0.9806		
Total	17.46	19			

0

kruskal-wallis

30

ANOVA table	SS	DF	MS	F (DFn, DF	P value
Treatment (between columns)	26.38	2	13.19	F (2, 16) =	P=0.0652
Residual (within columns)	64.84	16	4.053		

60

Total	91.23	18			
ANOVA table	SS	DF	MS	F (DFn, DF	P value
Treatment (between columns)	13.33	2	6.666	F (2, 16) =	P=0.0594
Residual (within columns)	31.5	16	1.968		
Total	44.83	18			

90

ANOVA table	SS	DF	MS	F (DFn, DF	P value
Treatment (between columns)	13.38	2	6.688	F (2, 16) =	P=0.0687
Residual (within columns)	33.65	16	2.103		
Total	47.03	18			

120

ANOVA table	SS	DF	MS	F (DFn, DF	P value
Treatment (between columns)	7.634	2	3.817	F (2, 16) =	P=0.1664
Residual (within columns)	30.38	16	1.899		
Total	38.02	18			

ox-LDL

C57

ANOVA table	SS	DF	MS	F (DFn, DF	P value
Treatment (between columns)	7.328	2	3.664	F (2, 18) =	P=0.6194
Residual (within columns)	134.1	18	7.449		
Total	141.4	20			

DB/DB

ANOVA table	SS	DF	MS	F (DFn, DF	P value
Treatment (between columns)	9.658	2	4.829	F (2, 17) =	P=0.2787
Residual (within columns)	59.54	17	3.502		
Total	69.2	19			

Y-MAZE

C57

ANOVA table	SS	DF	MS	F (DFn, DF	P value
Treatment (between columns)	0.03582	2	0.01791	F (2, 17) =	P=0.1817
Residual (within columns)	0.1612	17	0.009483		
Total	0.197	19			

DB/DB

ANOVA table	SS	DF	MS	F (DFn, DF	P value
Treatment (between columns)	0.002628	2	0.001314	F (2, 18) =	P=0.9231
Residual (within columns)	0.2942	18	0.01634		
Total	0.2968	20			

CRP

C57

ANOVA table	SS	DF	MS	F (DFn, DF	P value
Treatment (between columns)	2368	2	1184	F (2, 22) =	P=0.4049
Residual (within columns)	27649	22	1257		
Total	30017	24			

DB/DB

kruskal-wallis

IL-1A

C57

kruskal-wallis

DB/DB

ANOVA table	SS	DF	MS	F (DFn, DF	P value
Treatment (between columns)	3344	2	1672	F (2, 21) =	P=0.0250
Residual (within columns)	7946	21	378.4		
Total	11291	23			

TNF-A

C57

ANOVA table	SS	DF	MS	F (DFn, DF	P value
Treatment (between columns)	63635	2	31817	F (2, 19) =	P=0.0715
Residual (within columns)	198811	19	10464		
Total	262446	21			

DB/DB

kruskal-wallis
