# Supplementary material for: A novel palmitic acid hydroxy stearic acid (5‐PAHSA) plays a neuroprotective role by inhibiting phosphorylation of the m‐TOR‐ULK1 pathway and regulating autophagy
Source: CNS Neurosci Ther. 2021 Jan 18;27(4):484–96. doi: 10.1111/cns.13573 (PMC7941174; doi:10.1111/cns.13573)
Supplement: Supplementary file 3 — Supplementary Material [file CNS-27-484-s002.pdf]

Full unedited gel/blot for Figure 3A

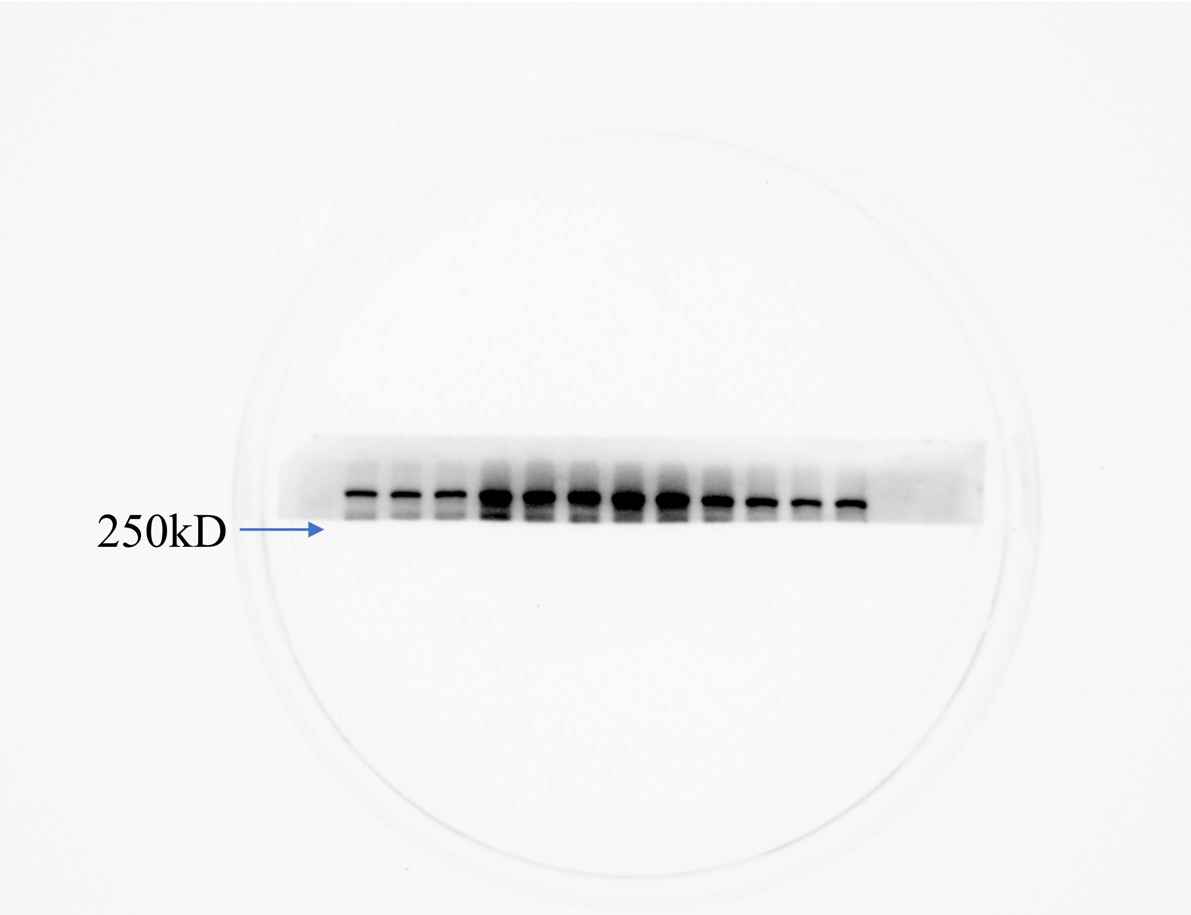

P-mTOR Ser 2448
(289kD)

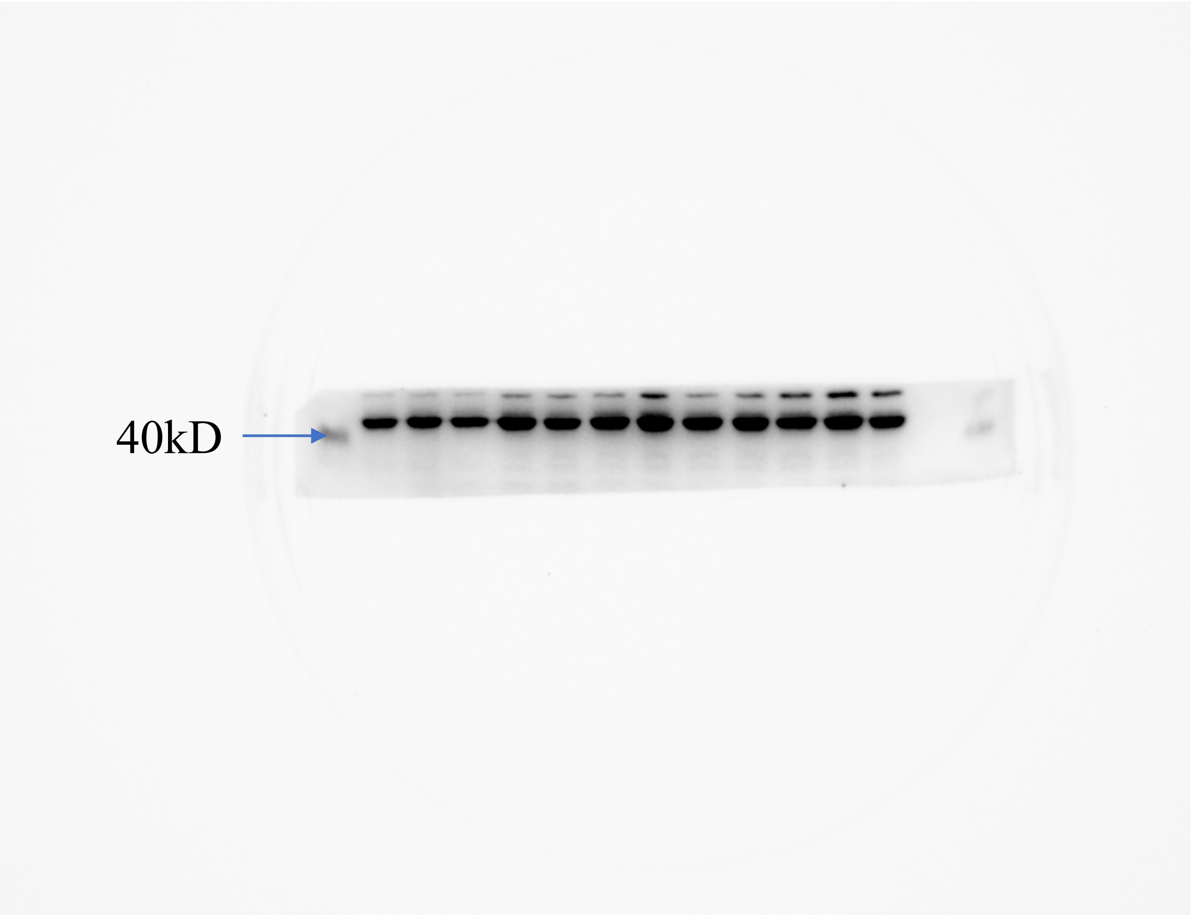

β-actin
(43kD)

Full unedited gel/blot for Figure 3B

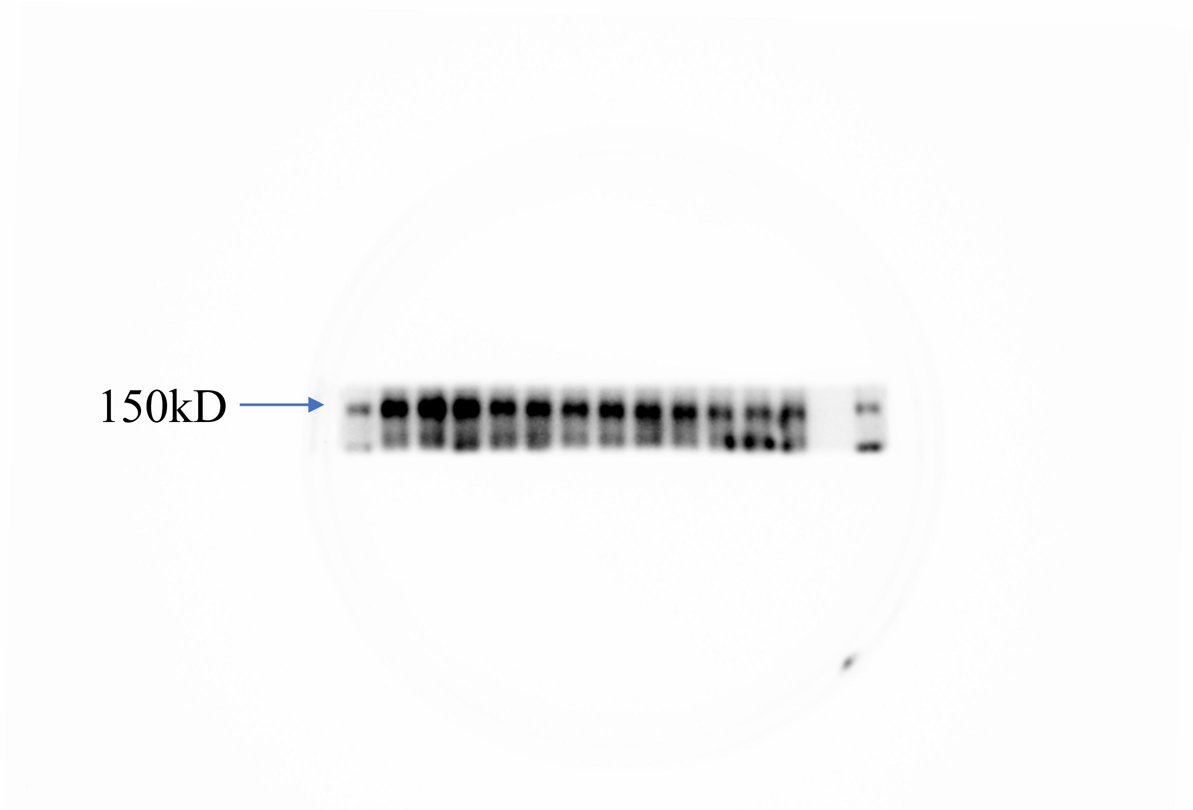

P-ULK-1 Ser 757
(150kD)

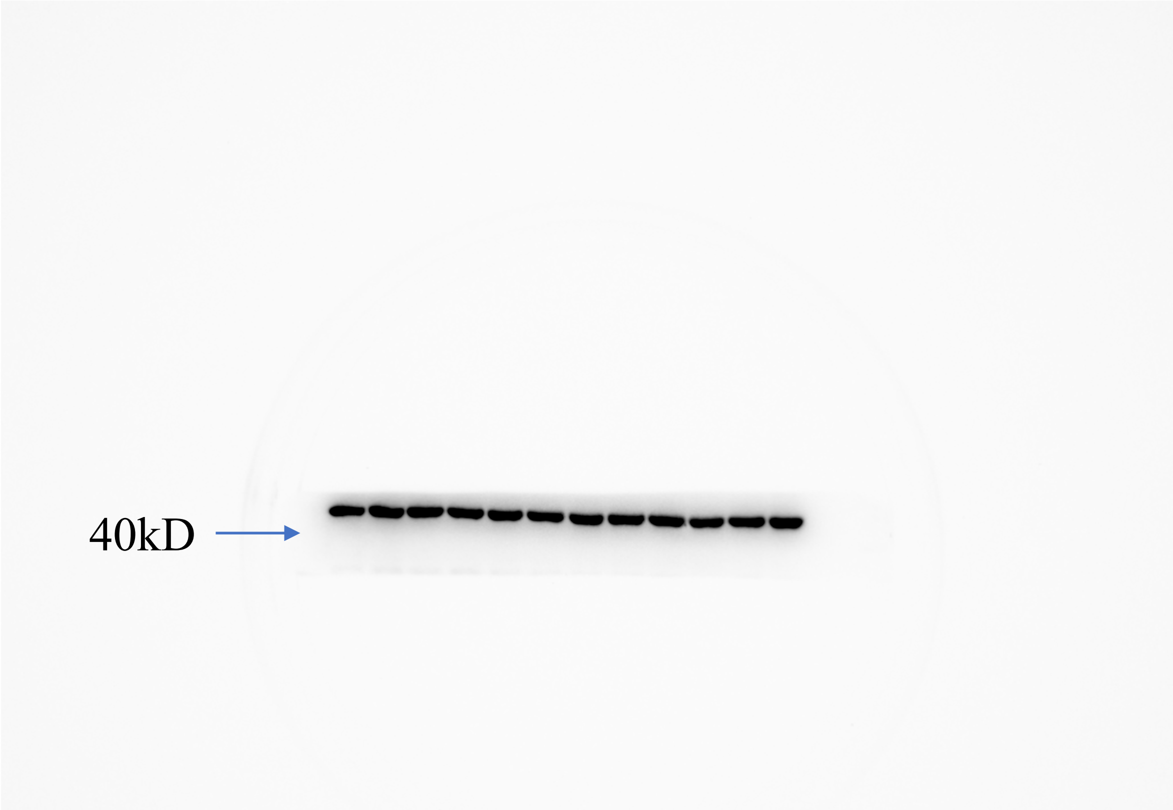

β-actin
(43kD)

Full unedited gel/blot for Figure 3C

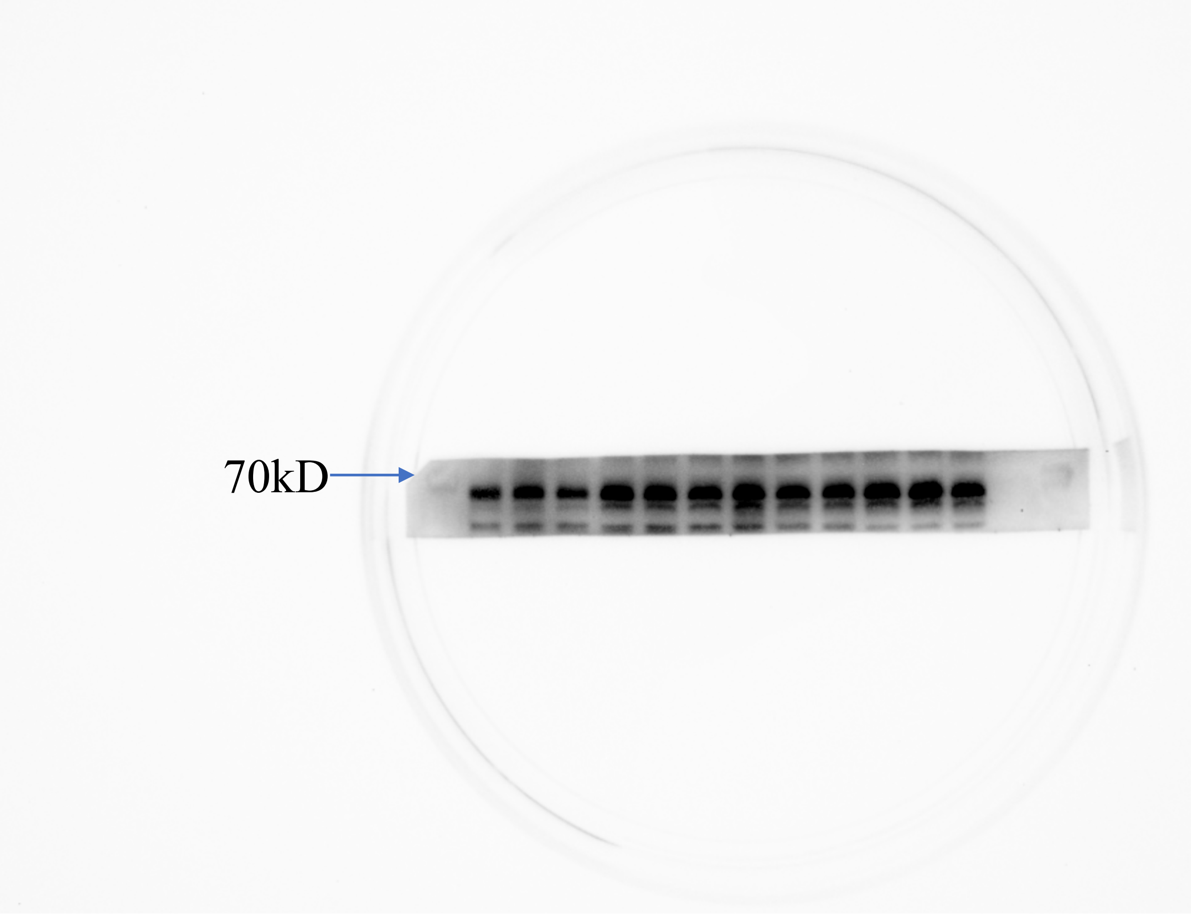

Beclin-1
(43kD)

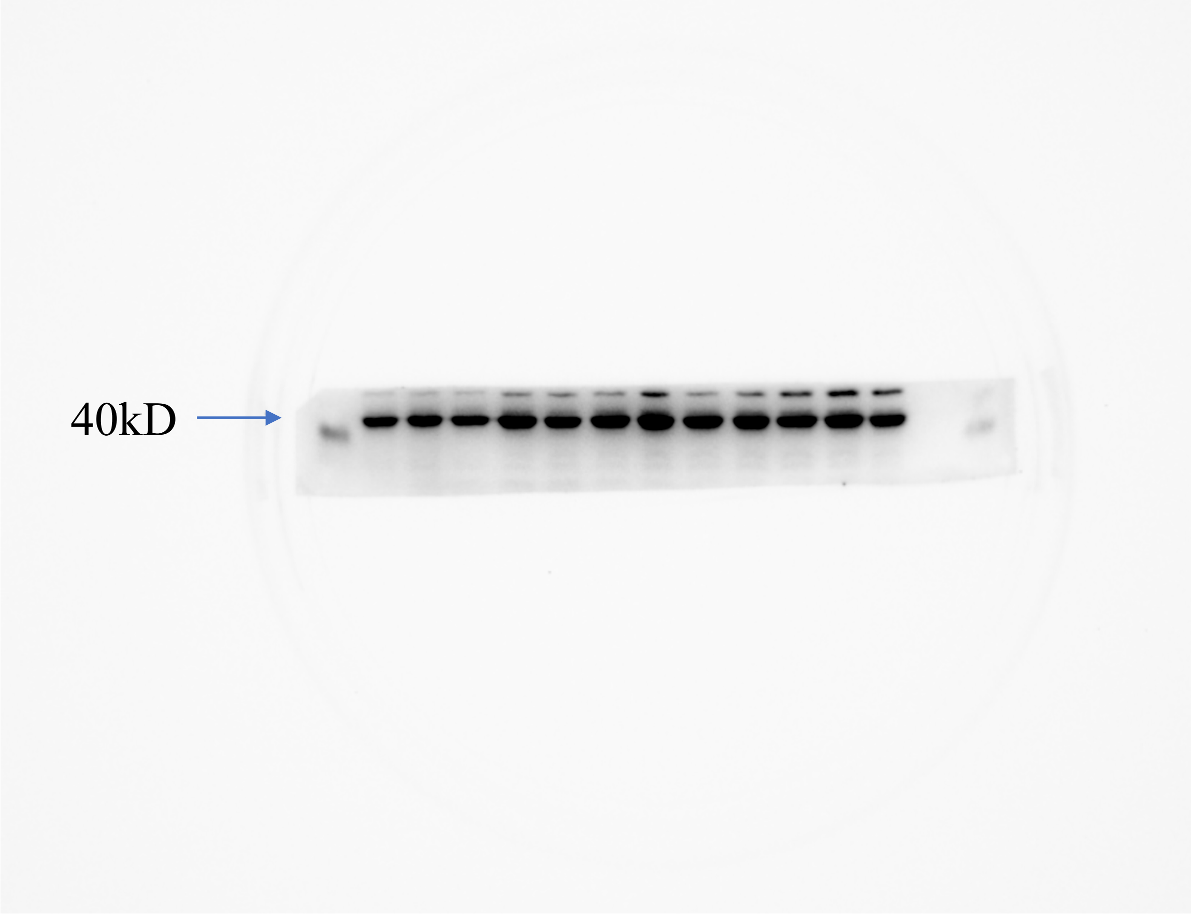

β-actin
(43kD)

Full unedited gel/blot for Figure 3D

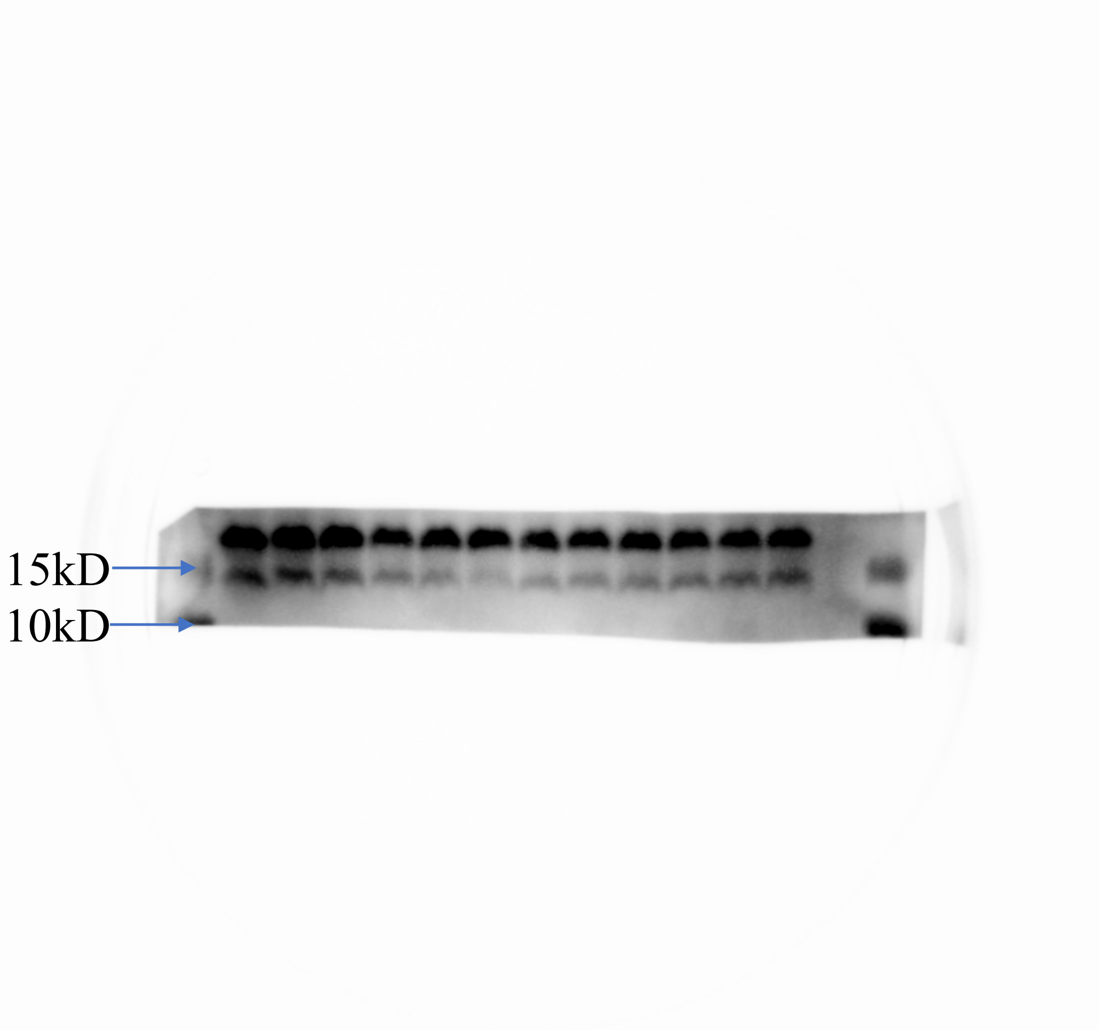

LC3 I (17kD)
LC3 II(14kD)

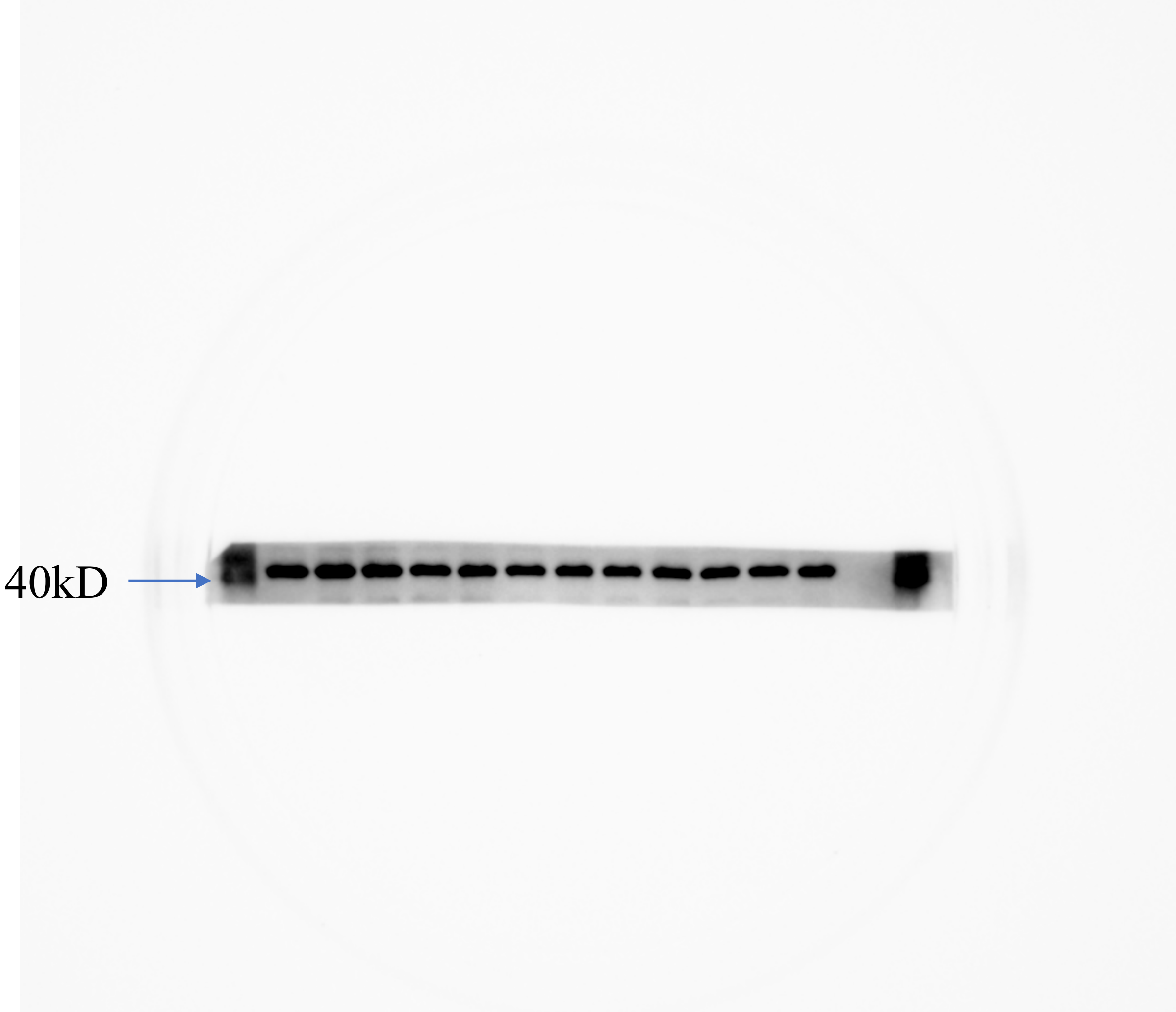

β-actin
(43kD)

Full unedited gel/blot for Figure 3E

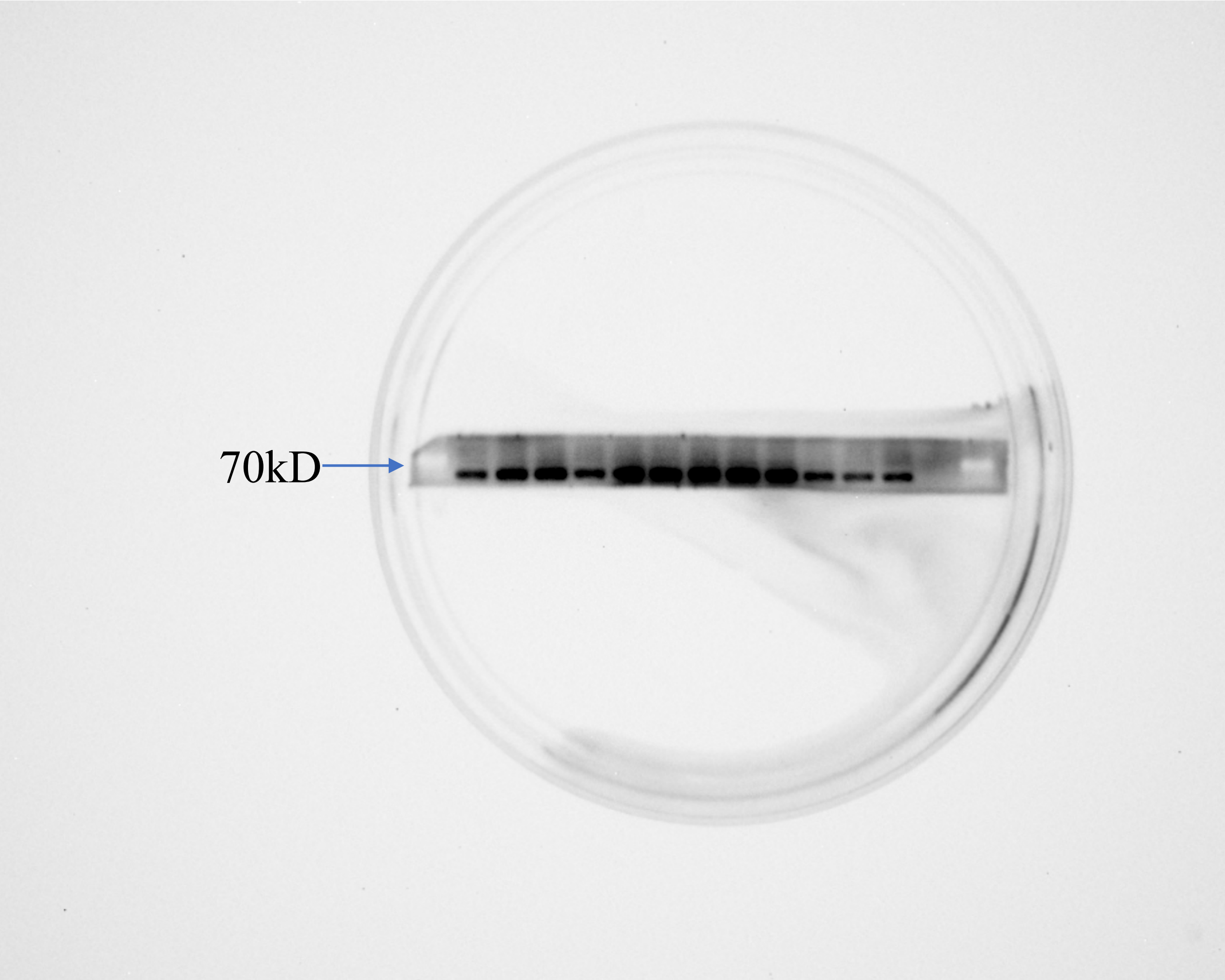

P62/SQSTM
(62kD)

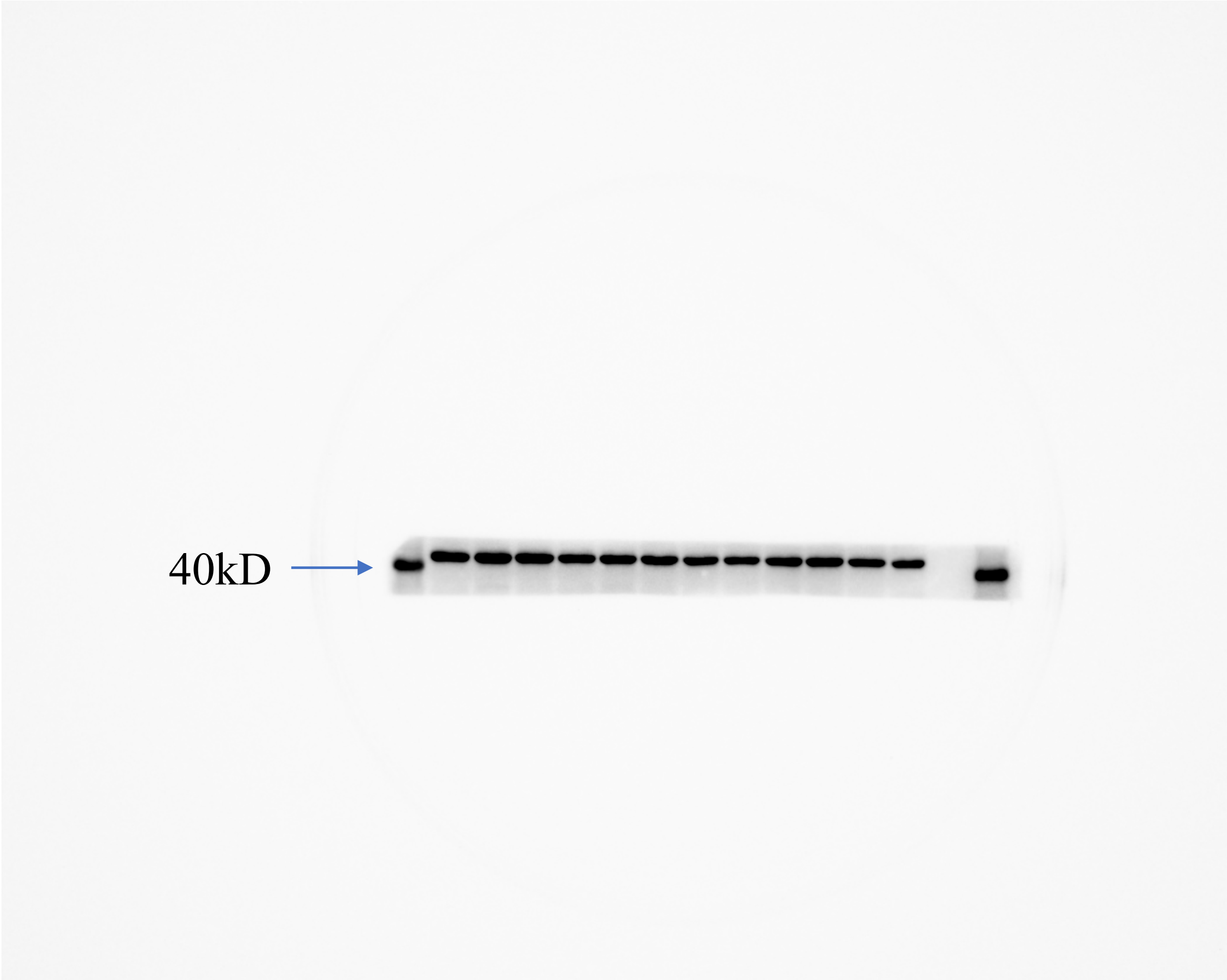

β-actin
(43kD)

Full unedited gel/blot for Figure 3F

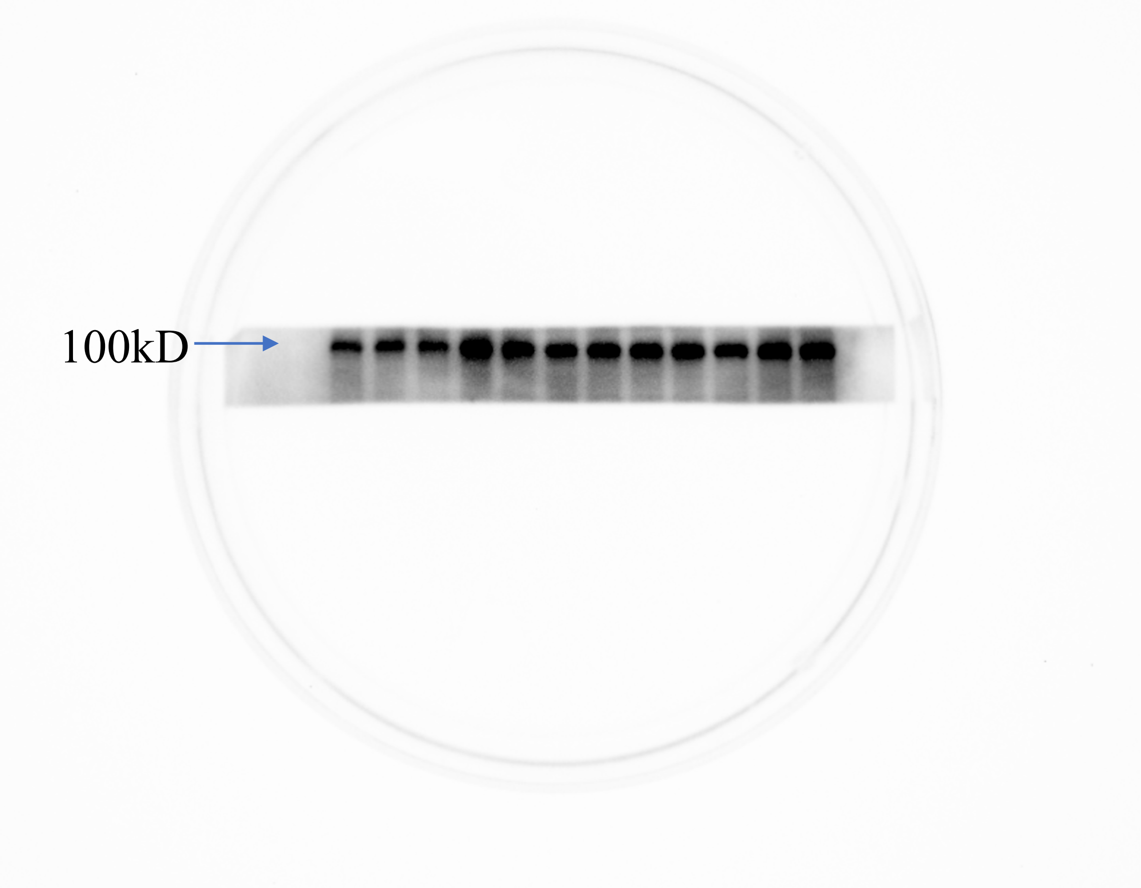

PI3K CIII(100kD)

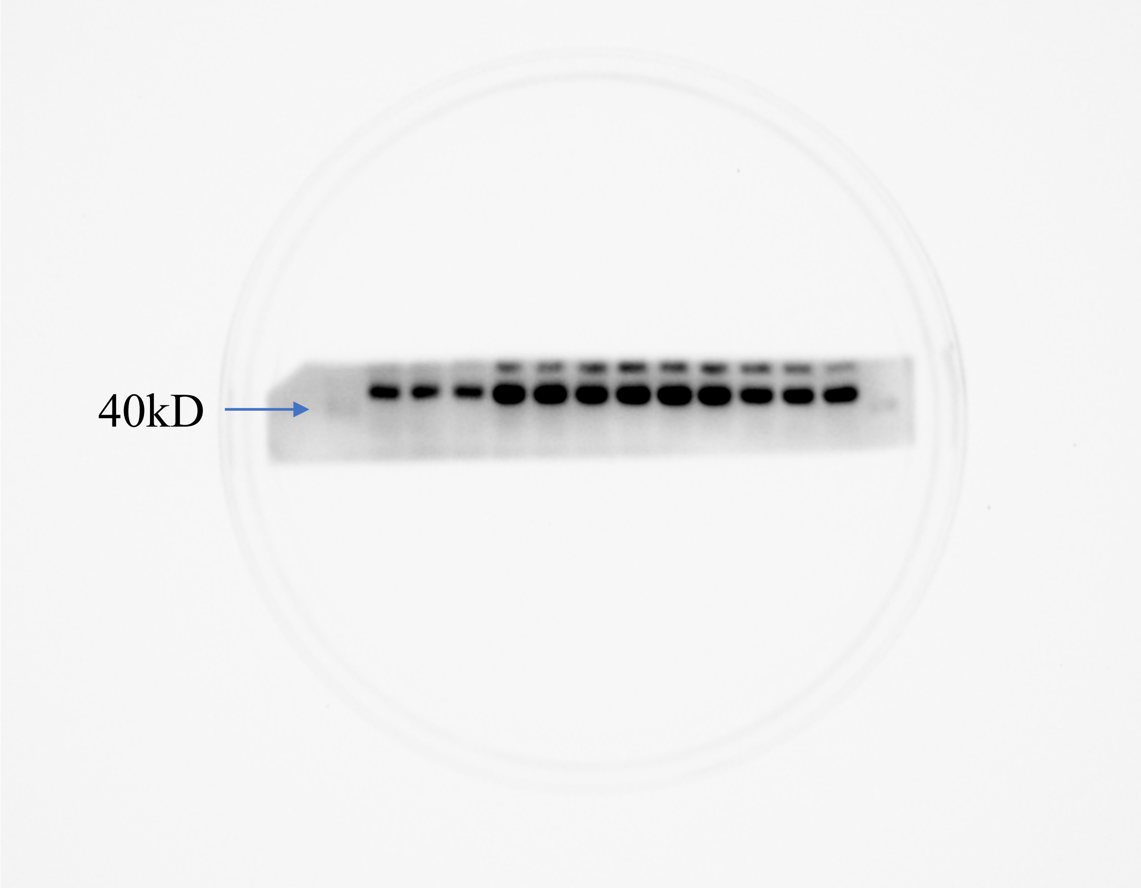

β-actin
(43kD)

Full unedited gel/blot for Figure 4A

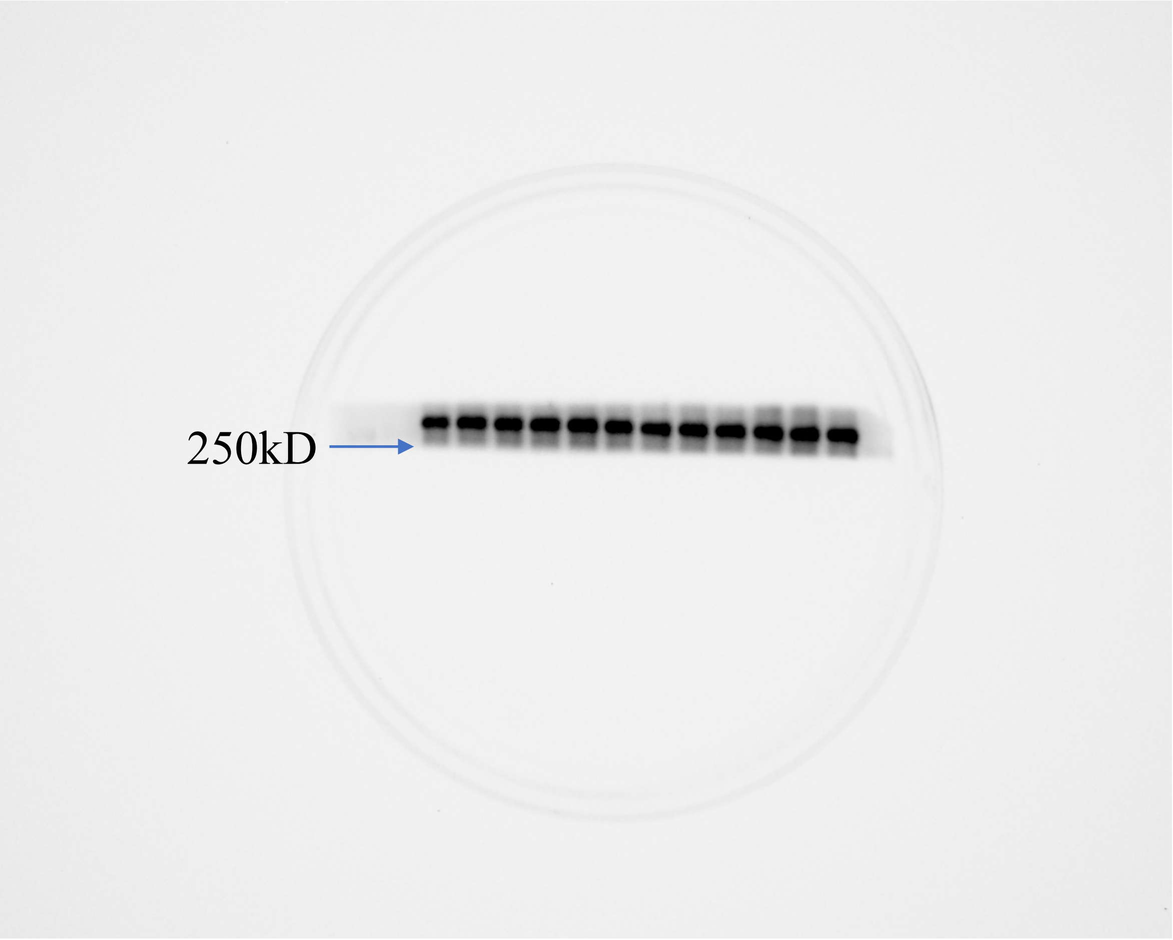

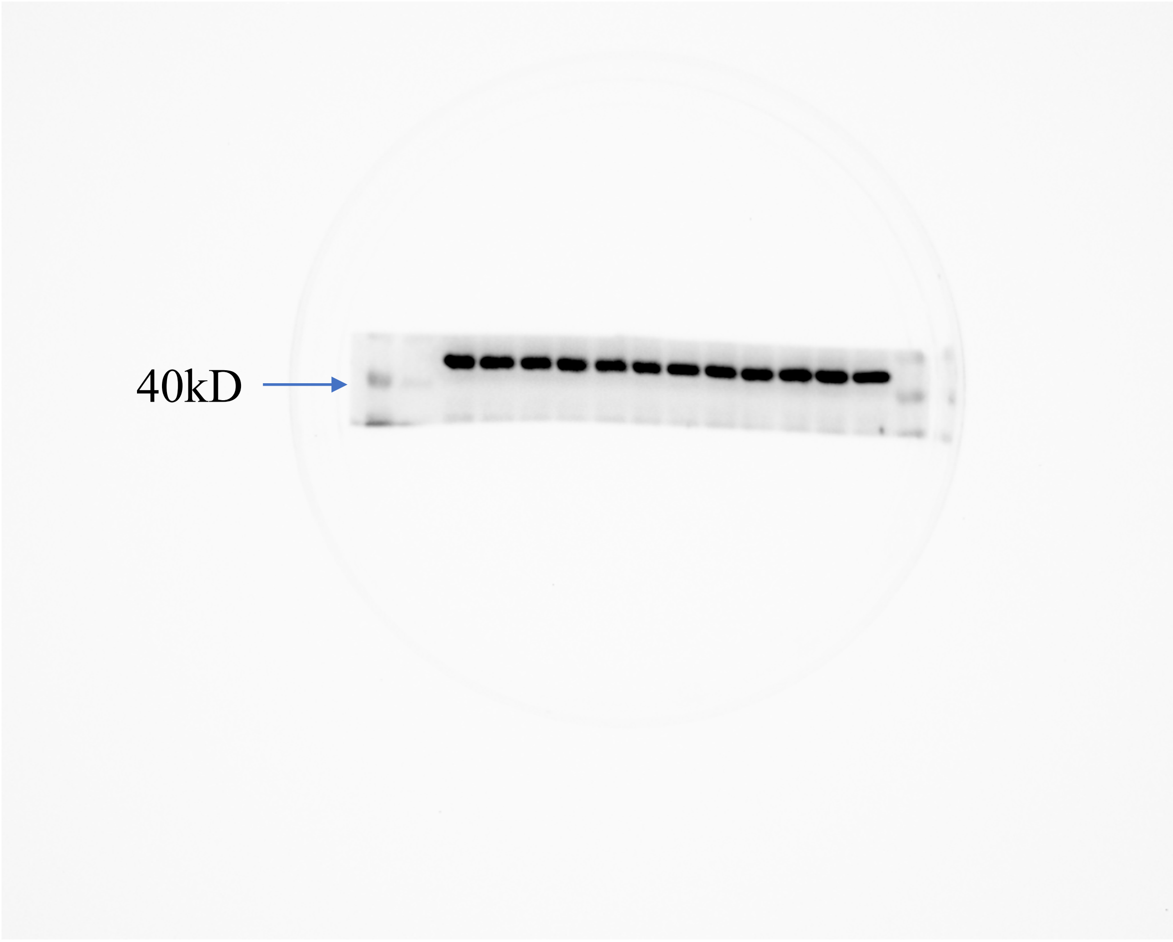

P-mTOR Ser 2448
(289kD)

β-actin
(43kD)

Full unedited gel/blot for Figure 4B

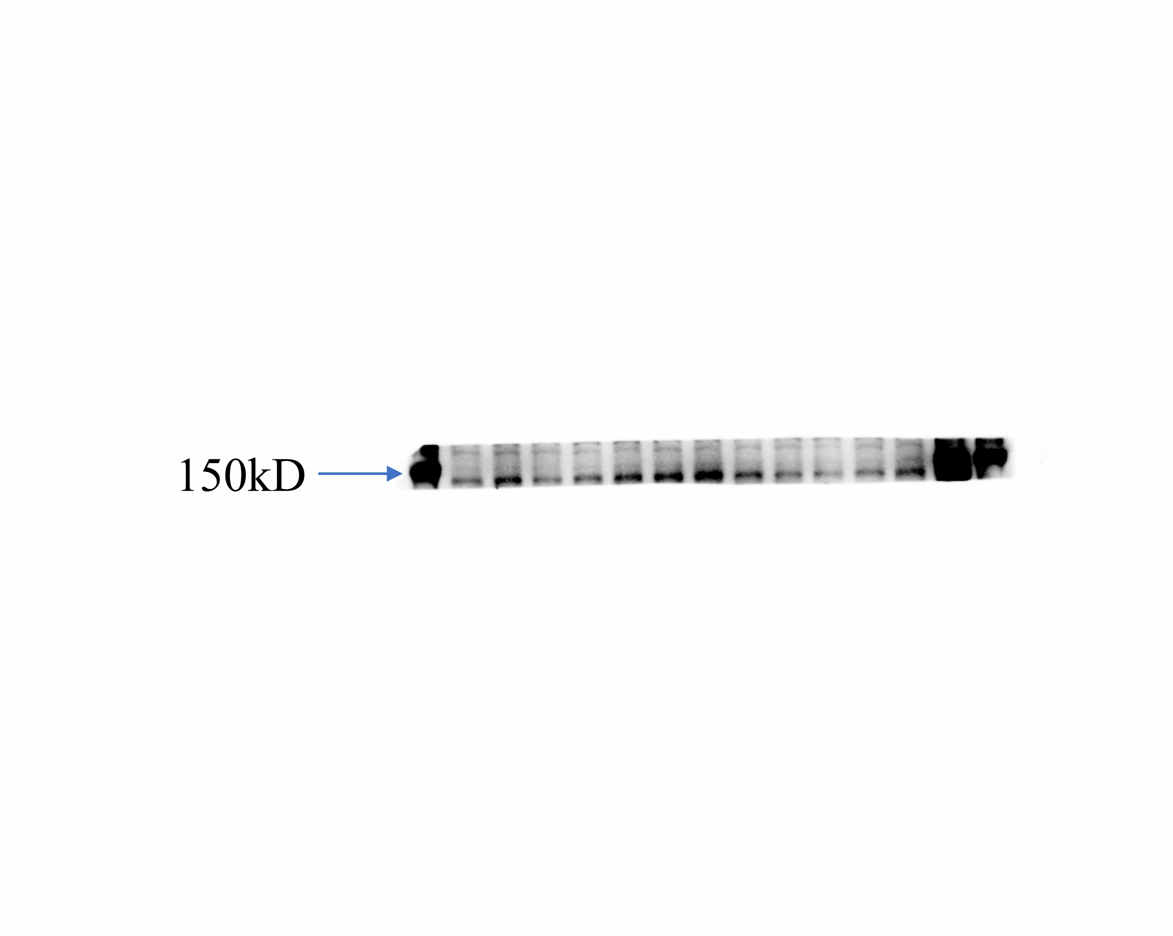

P-ULK-1 Ser 757
(150kD)

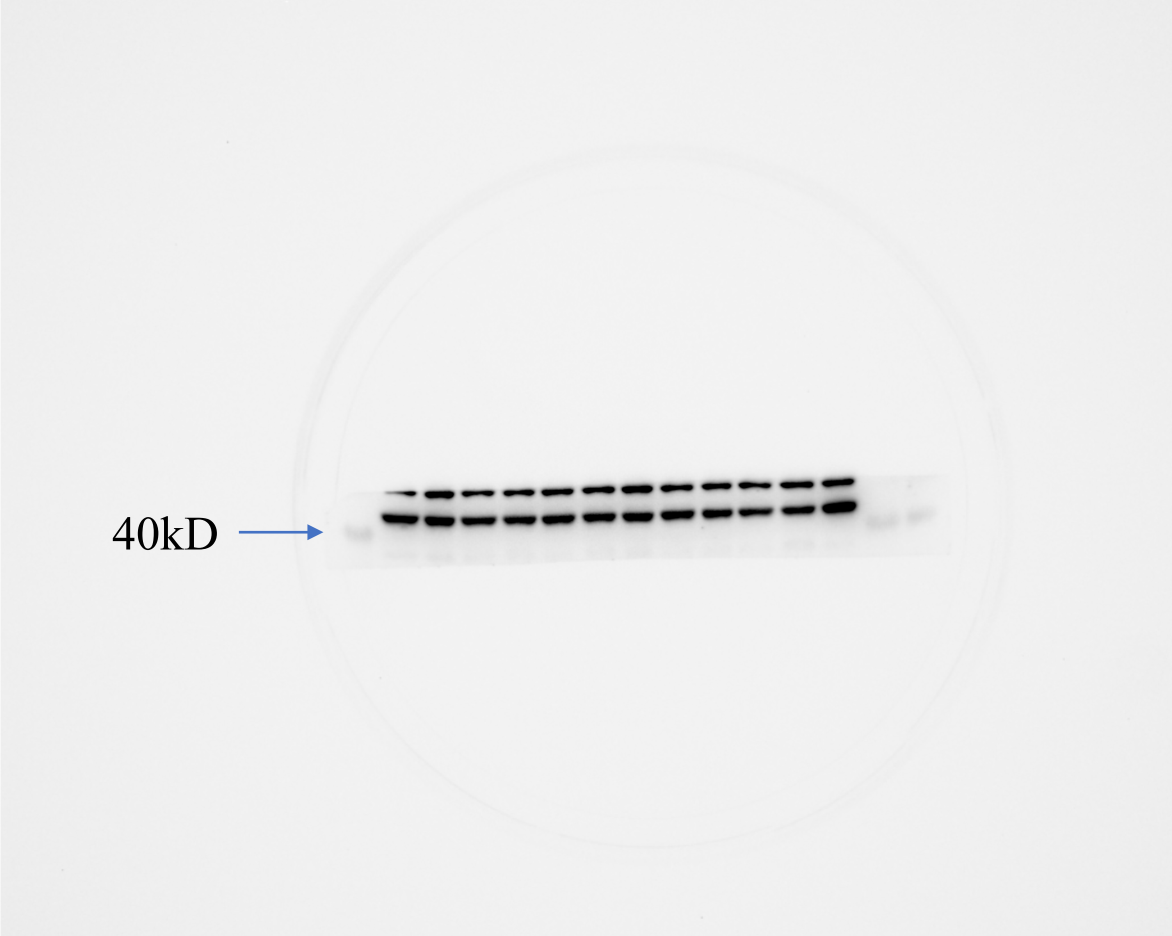

β-actin
(43kD)

Full unedited gel/blot for Figure 4C

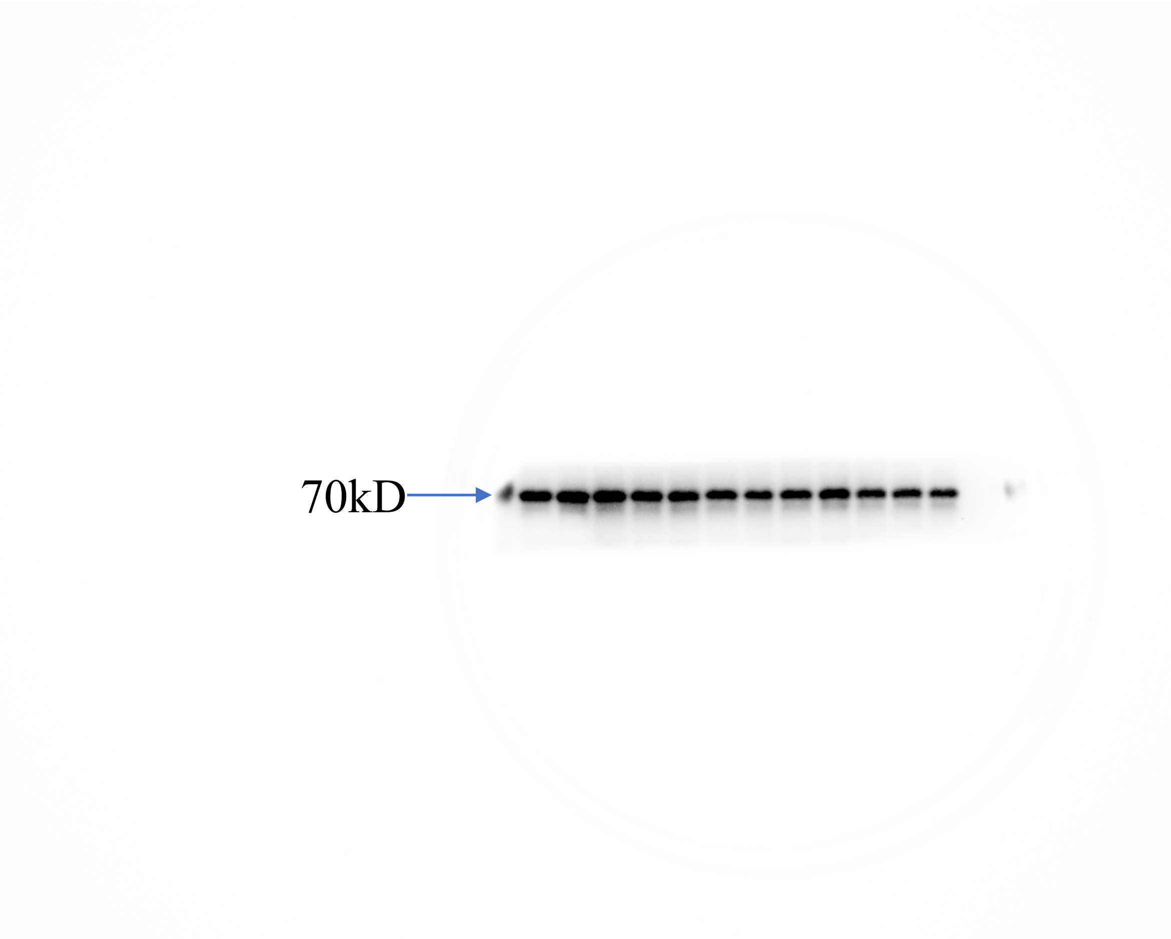

Beclin-1
(60kD)

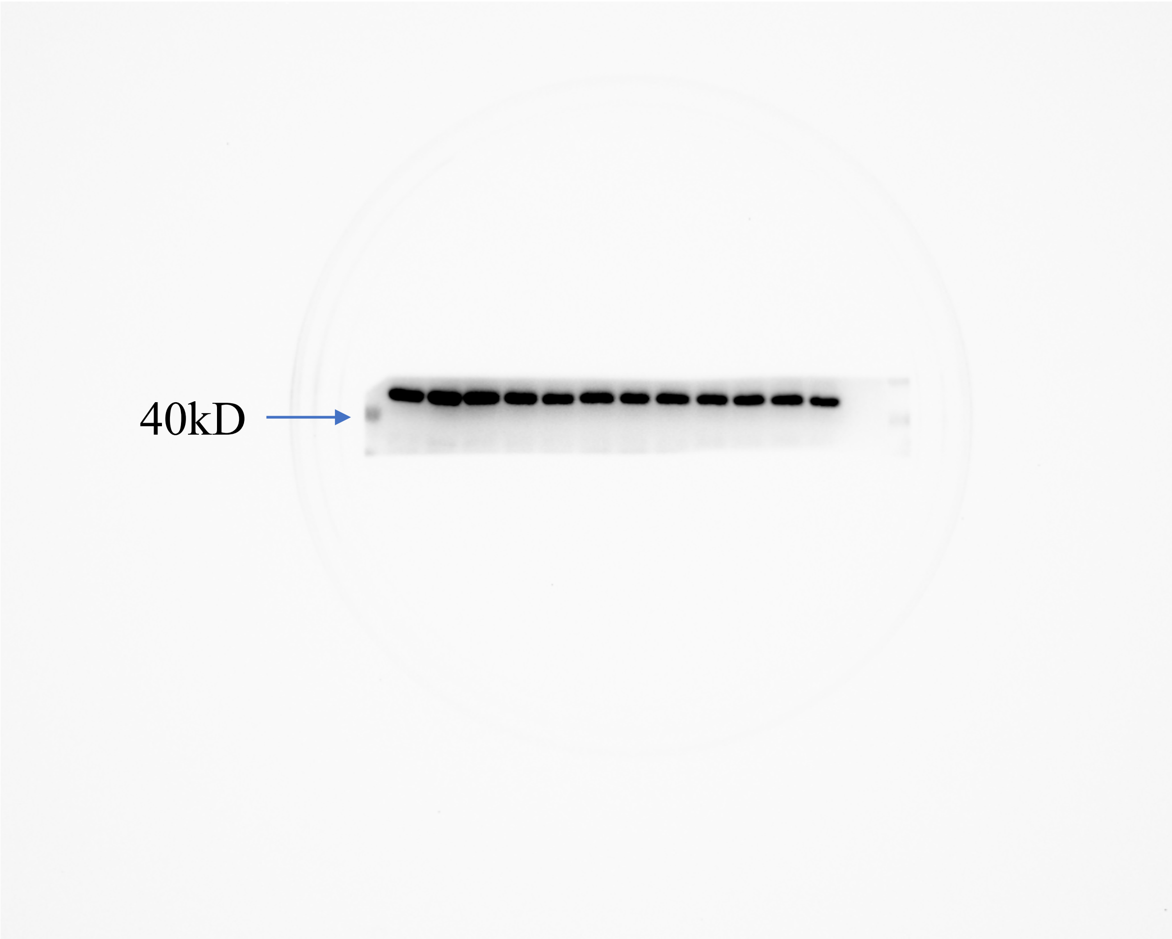

β-actin
(43kD)

Full unedited gel/blot for Figure 4D

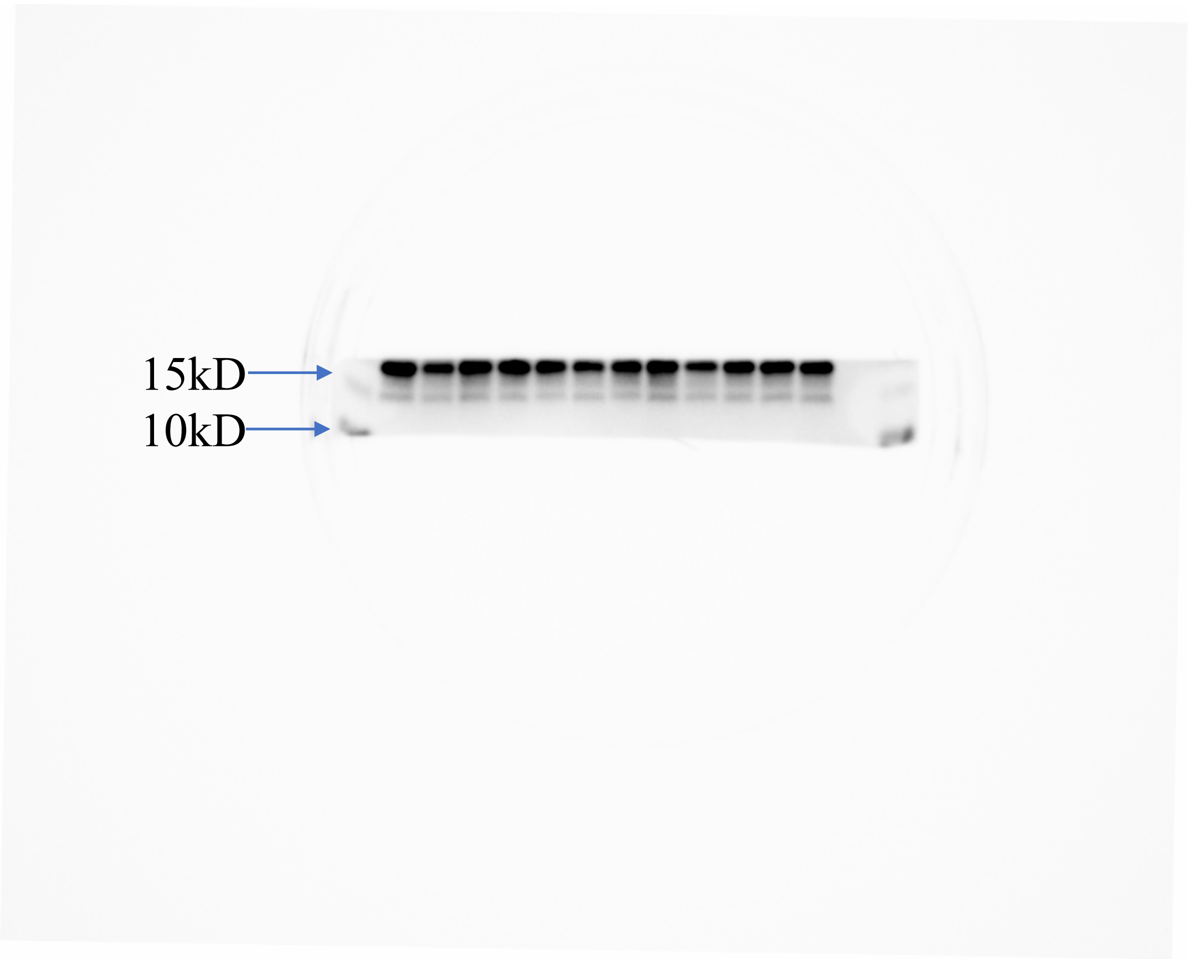

LC3 I (17kD)
LC3 II(14kD)

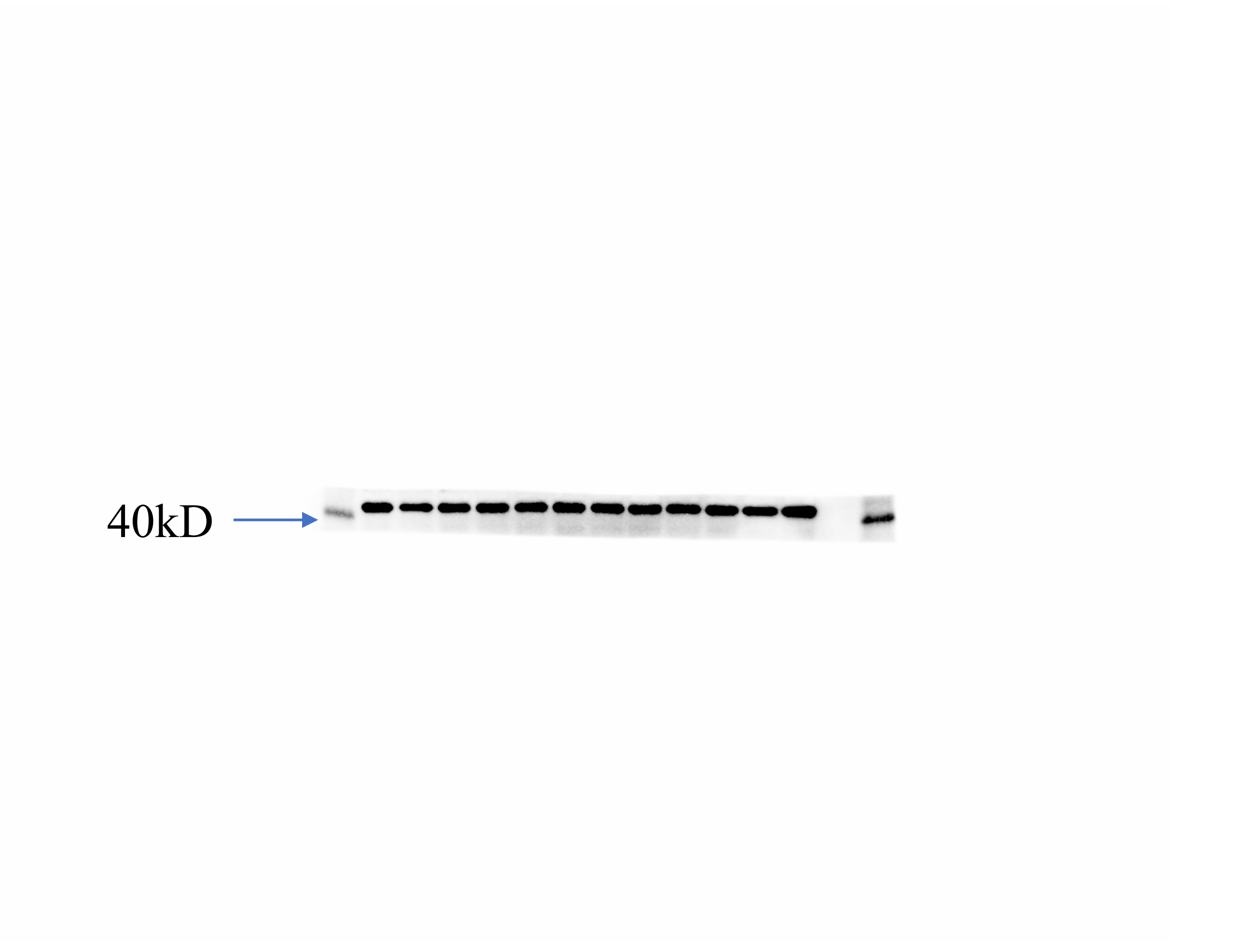

β-actin
(43kD)

Full unedited gel/blot for Figure 4E

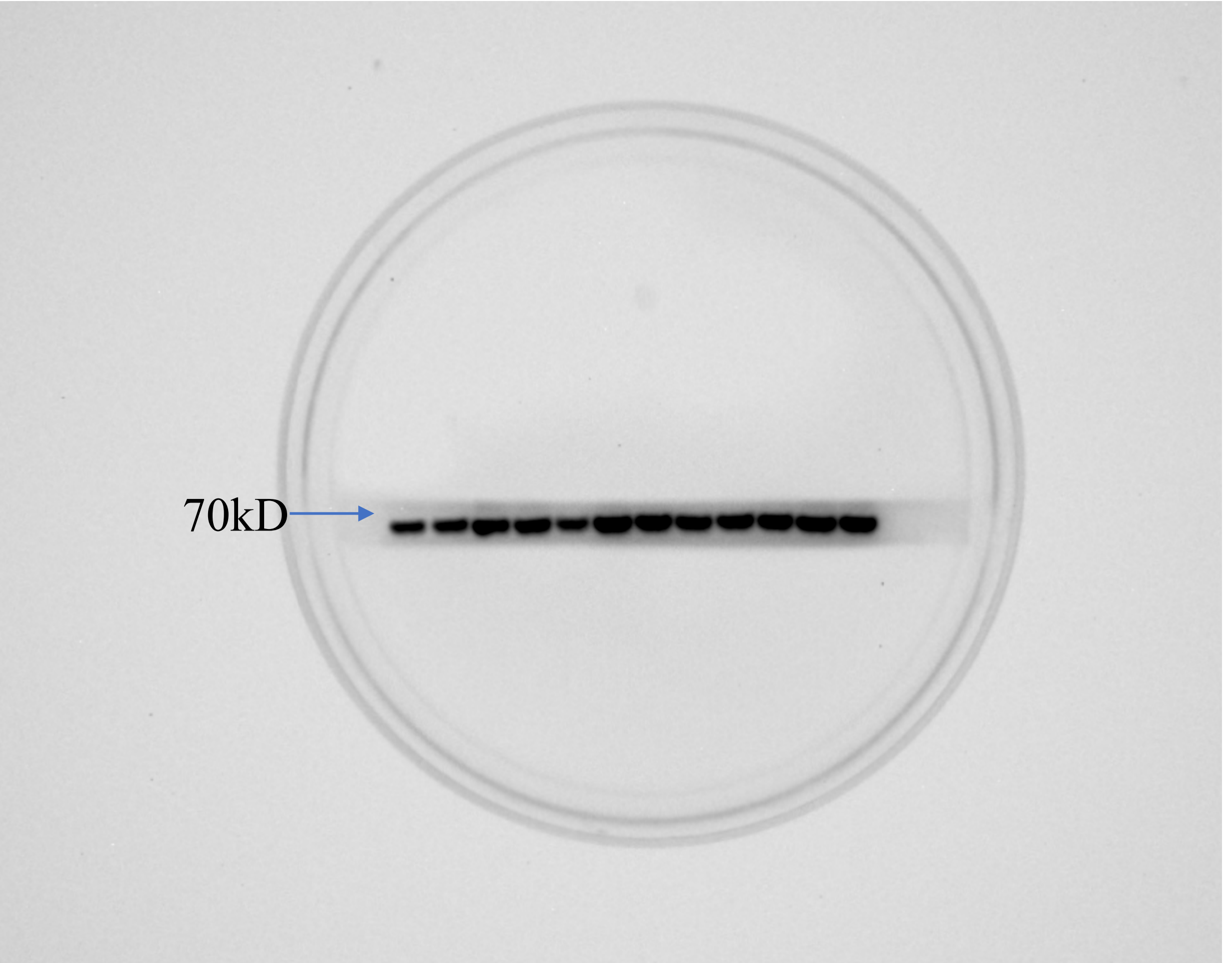

P62/SQSTM
(62kD)

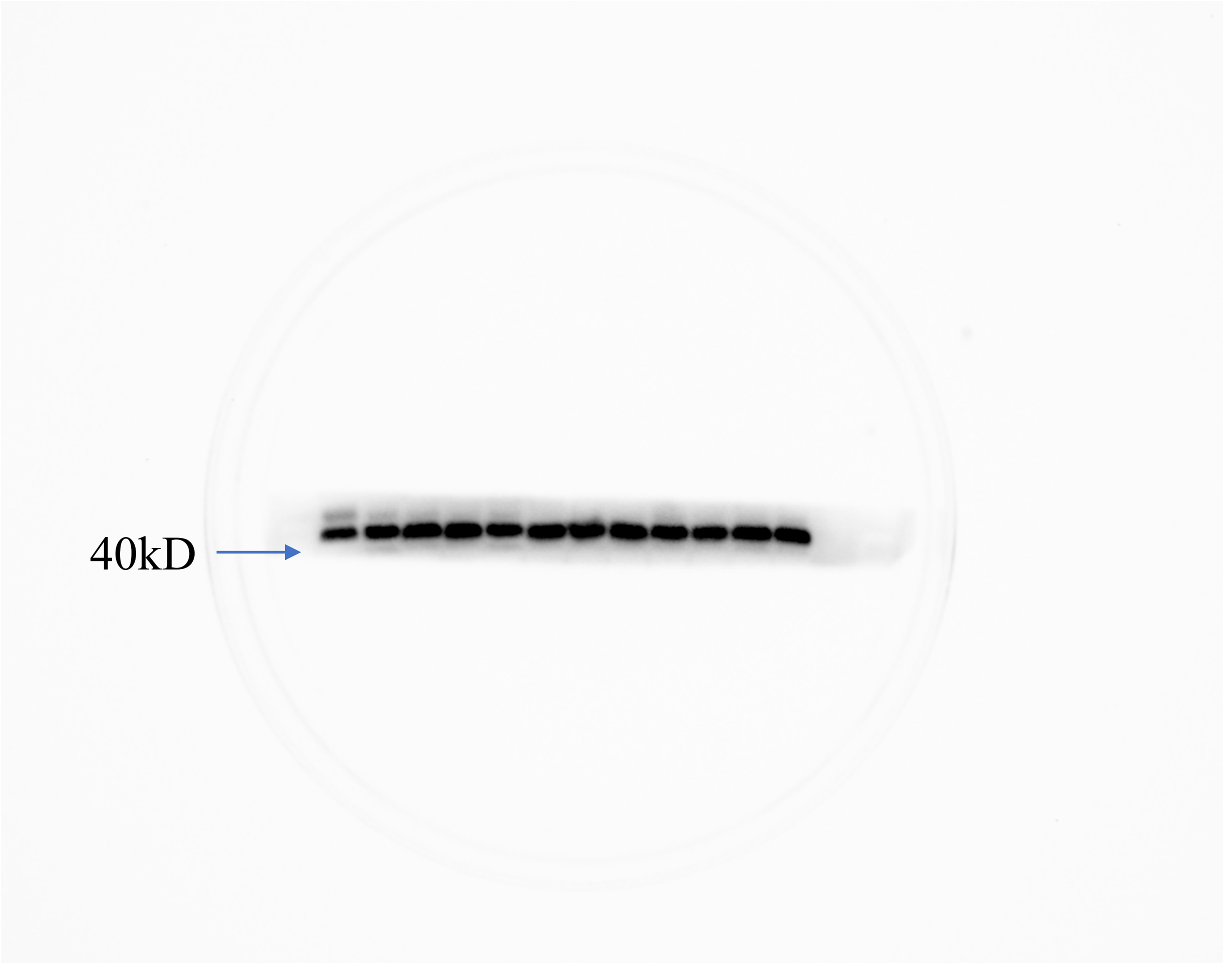

β-actin
(43kD)

Full unedited gel/blot for Figure 4F

100kD

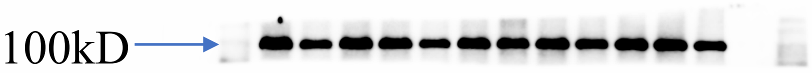

PI3K CIII(100kD)

40kD

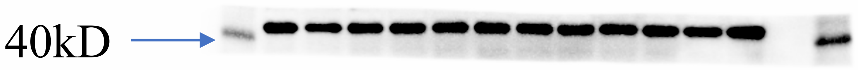

β-actin
(43kD)

Full unedited gel/blot for Figure 5A

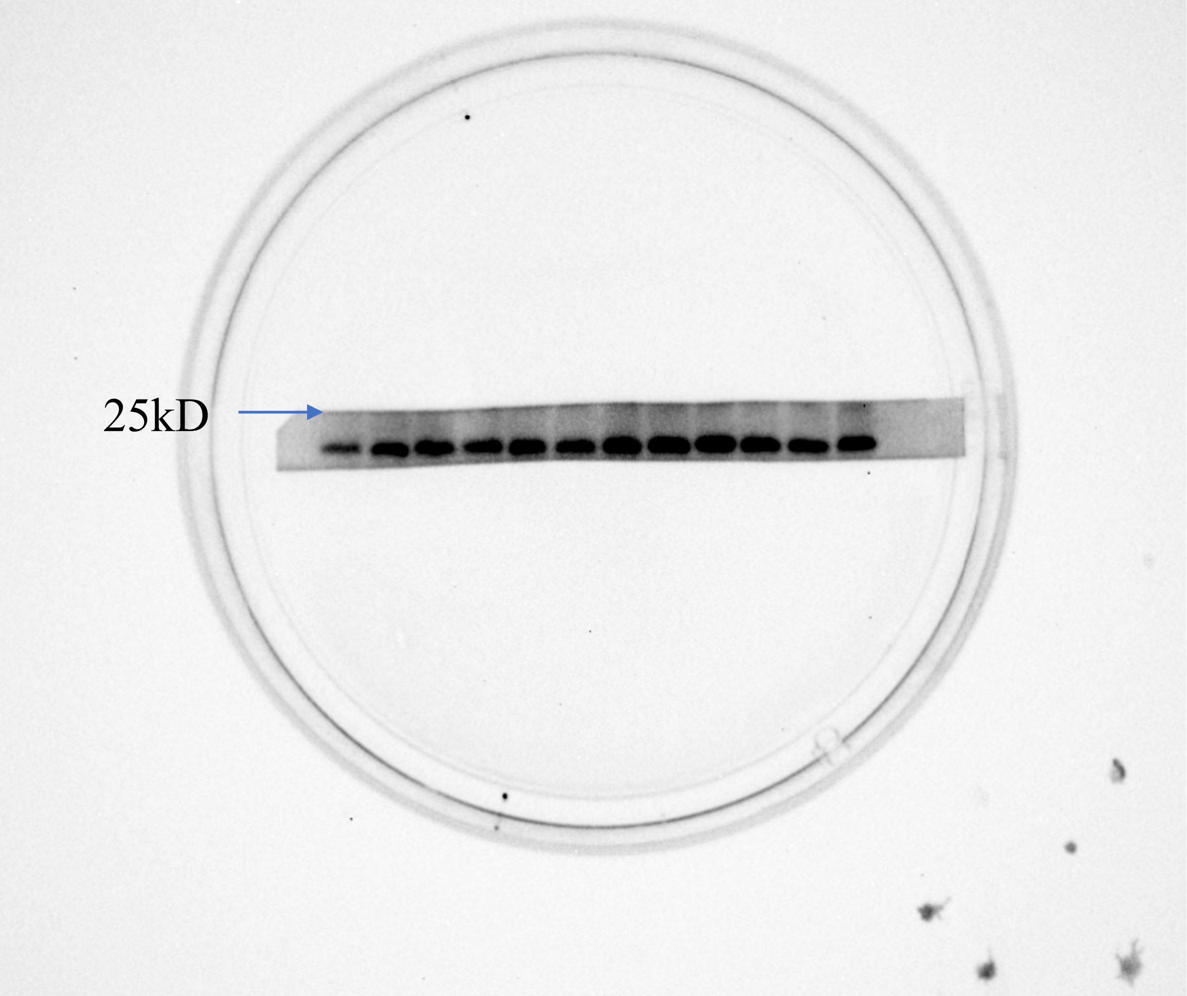

Bax (21kD)

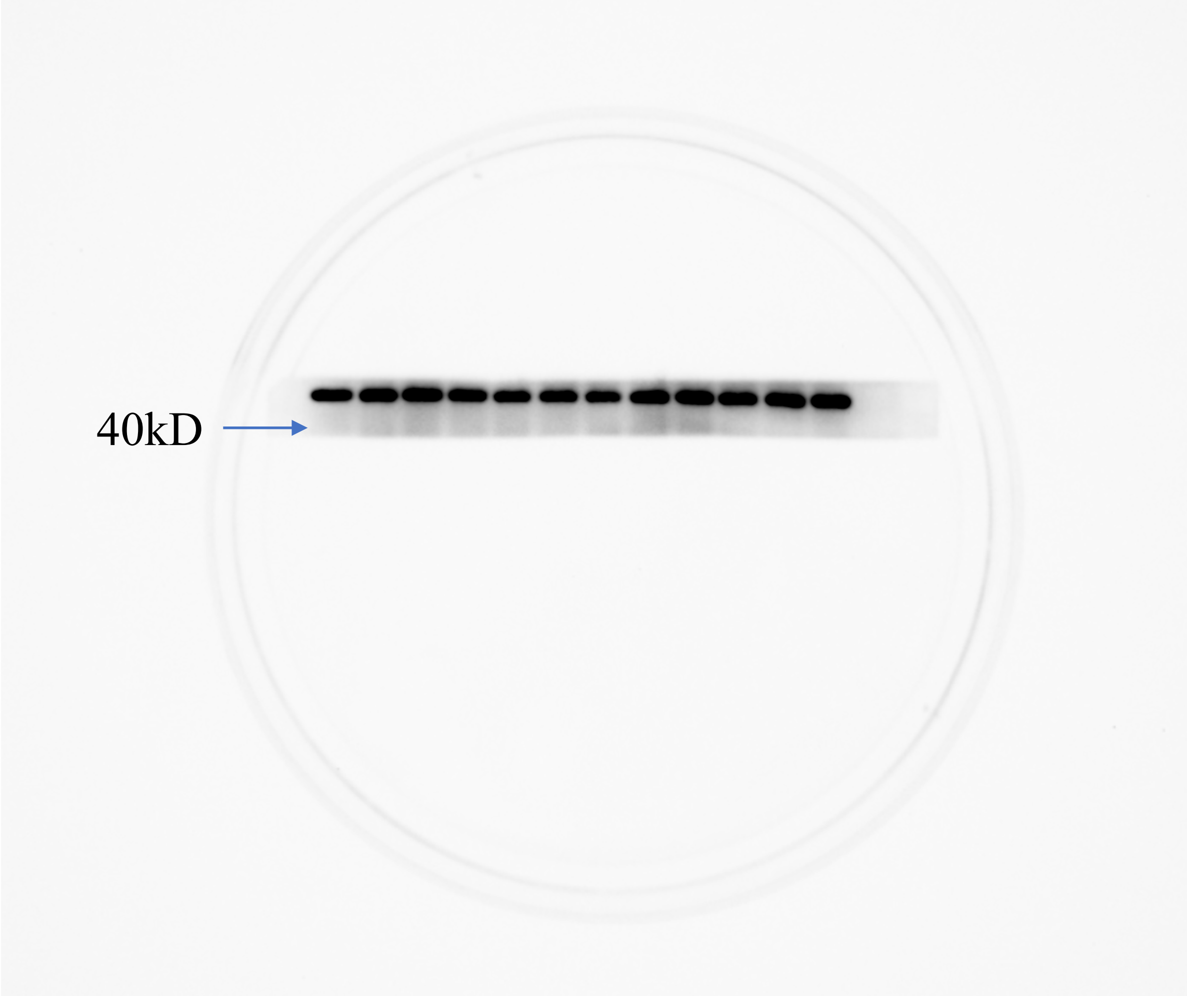

β-actin
(43kD)

Full unedited gel/blot for Figure 5B

15kD
10kD

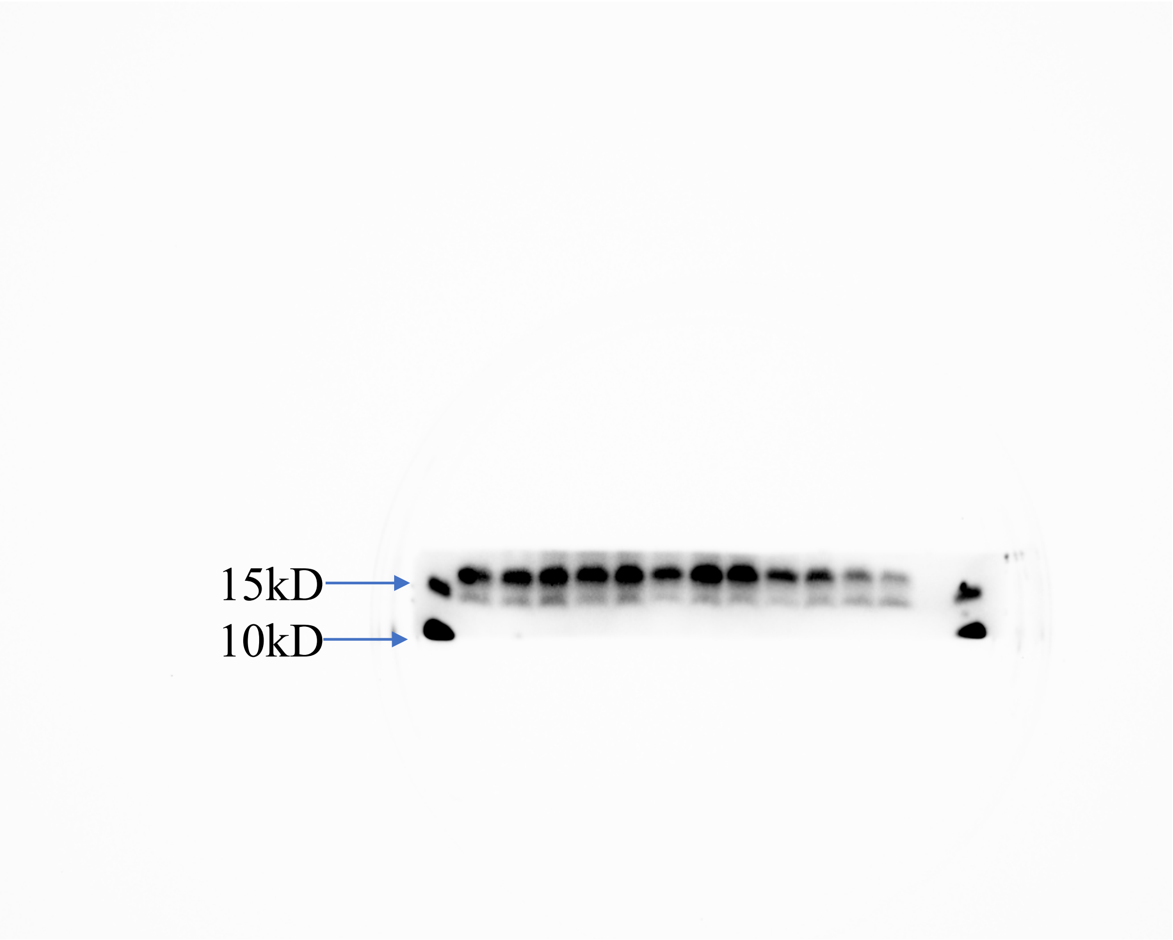

Cleaved caspase-3 (17kD)

40kD

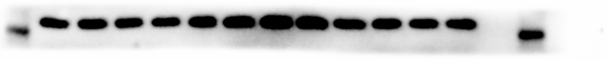

β-actin
(43kD)

Full unedited gel/blot for Figure 5C

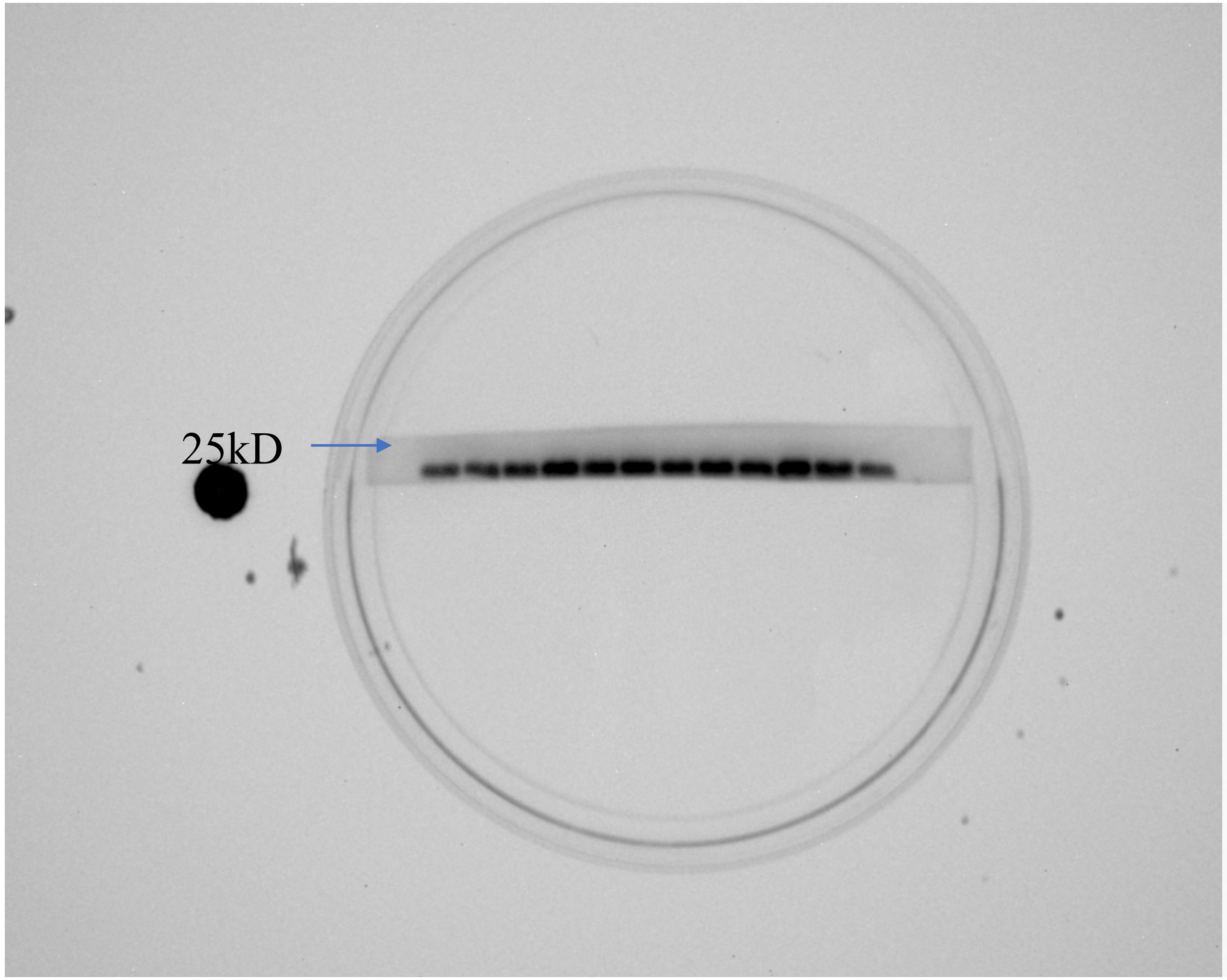

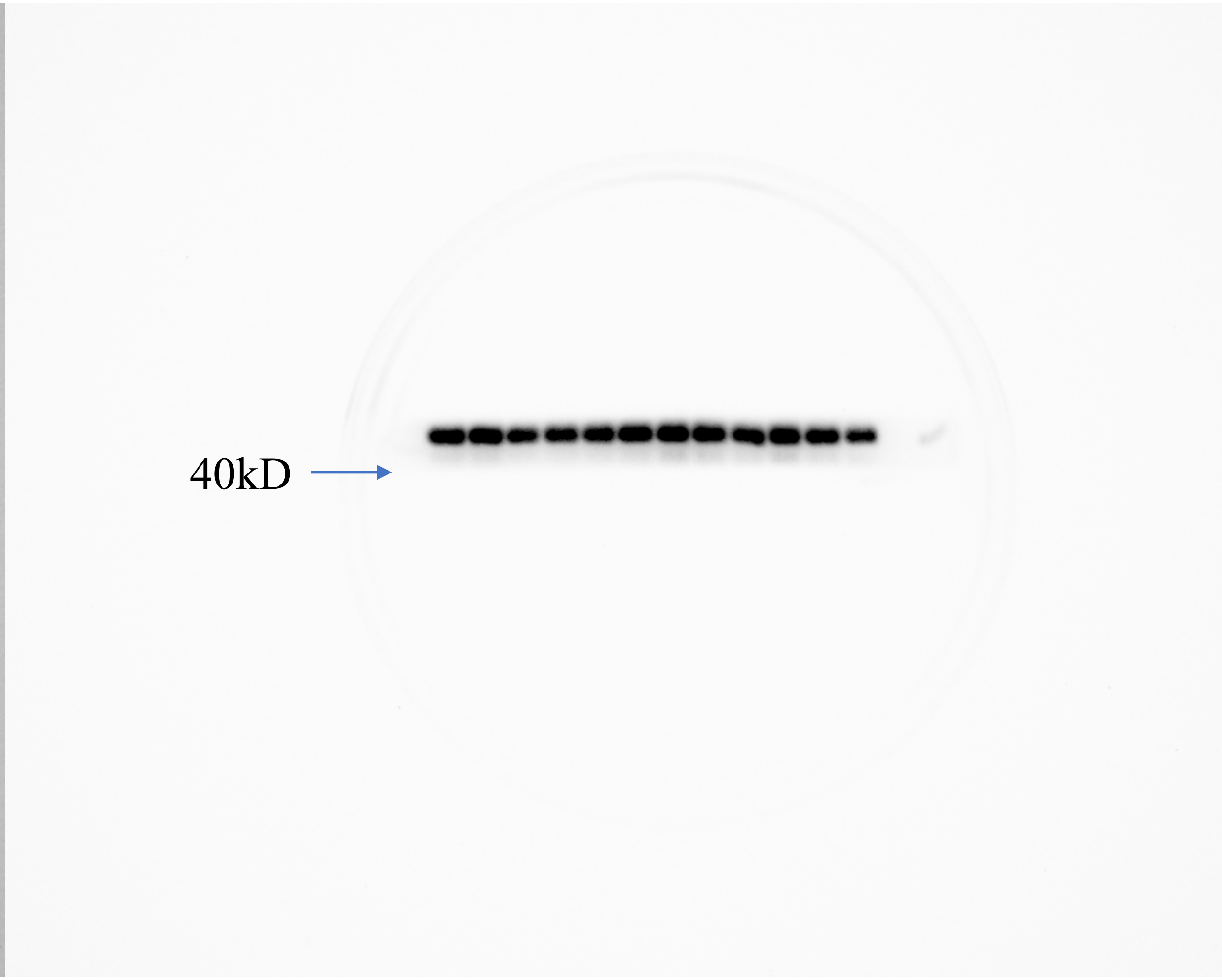

Bax (21kD)

β-actin
(43kD)

Full unedited gel/blot for Figure 5D

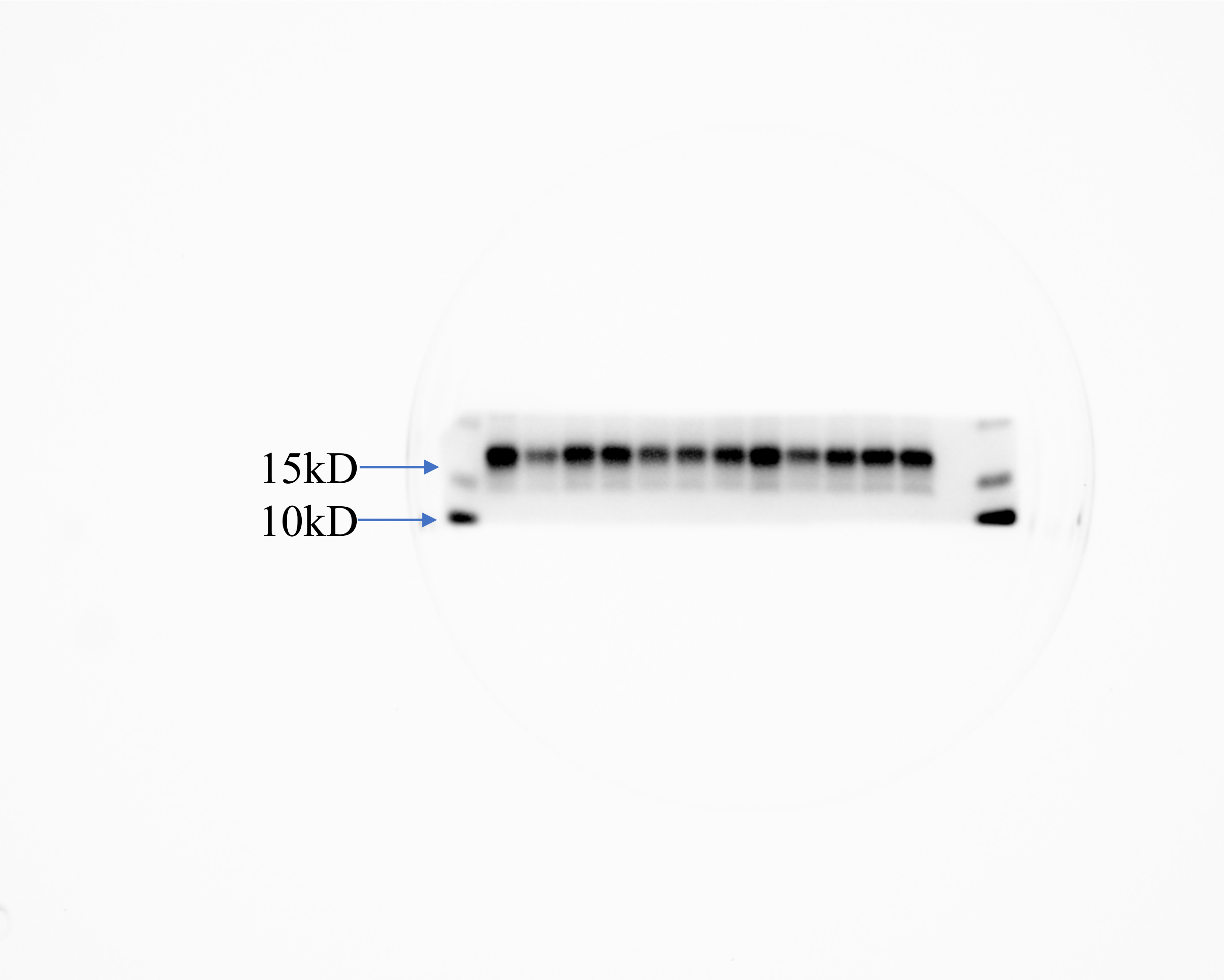

Cleaved caspase-3 (17kD)

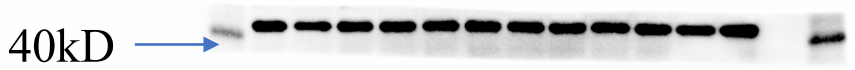

β-actin
(43kD)
